# CHAC1-linked NRF2 protein expression participates in pancreatic islet protection by dextrorphan

**DOI:** 10.1016/j.isci.2026.117474

**Published:** 2026-09-07

**Authors:** Celine Weippert, Philip Kirschner, Barbara Bartosinska, Andrea Köster, Daniel Eberhard, Eckhard Lammert

**Affiliations:** 1 Institute of Metabolic Physiology, Heinrich Heine University, 40225 Düsseldorf, Germany; 2 Institute for Vascular and Islet Cell Biology, German Diabetes Center (DDZ), Leibniz Center for Diabetes Research at Heinrich Heine University, 40225 Düsseldorf, Germany; 3 German Center for Diabetes Research (DZD e.V.), Helmholtz Zentrum München, 85764 Neuherberg, Germany

**Keywords:** pancreatic beta cell, beta-cell survival, cellular stress response, redox homeostasis

## Abstract

The N-methyl-D-aspartate (NMDA) receptor antagonist dextrorphan (DXO) protects pancreatic islets from cell death induced by streptozotocin (STZ), inflammatory cytokines, reactive oxygen species (ROS), as well as in multiple mouse models of diabetes. Here we show that cytoprotective concentrations of DXO upregulate the transcription factor nuclear factor erythroid 2-related factor 2 (NRF2), driving the expression of its downstream target genes. Paradoxically, DXO also increases the expression of the pro-apoptotic factor CHAC1 (chaC glutathione-specific γ-glutamylcyclotransferase 1). To investigate its role, we silenced *Chac1* in islets, which significantly enhanced DXO-mediated cell protection. In contrast, both pharmacological inhibition and genetic knockdown of *Nfe2l2* diminished the protective effects of DXO, confirming the cytoprotective role of NRF2. Conversely, pharmacological activation of NRF2 did not further enhance DXO-induced protection. Together, our data indicate that while DXO-mediated islet protection requires induction of NRF2, its full therapeutic efficacy is limited by the simultaneous upregulation of CHAC1.

## Highlights

• NMDA receptor antagonist DXO induces NRF2 target genes and *Chac1* expression
• DXO saves NRF2 protein from breakdown in pancreatic islets
• CHAC1 partially counteracts the islet cell-protective effects of DXO
• DXO requires NRF2 to successfully prevent pancreatic islet cell death

## Graphical abstract

**Figure:**
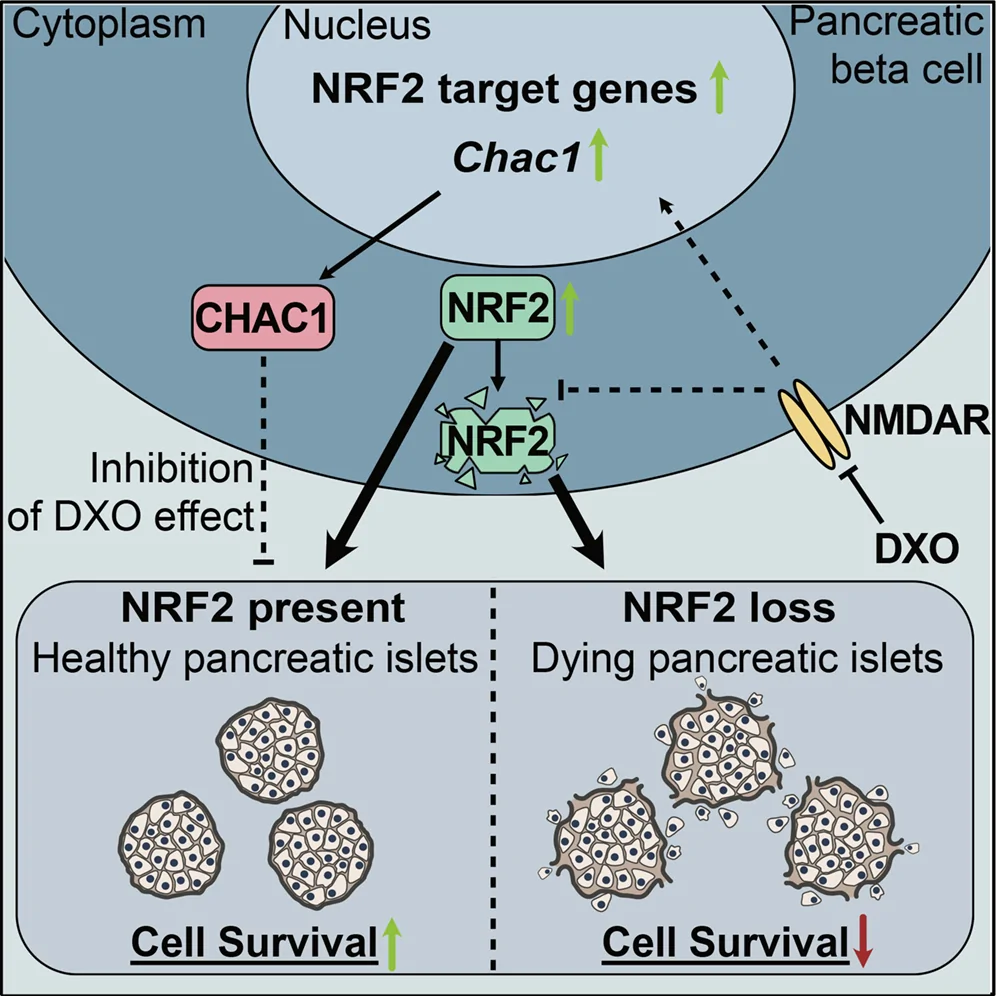

## Teaser

Molecular biology; Molecular interaction; Molecular network

## Introduction

Diabetes mellitus (DM) is a metabolic disorder characterized by persistent hyperglycemia caused by inadequate insulin secretion, impaired insulin response, or both.^1^^,^^2^ Over time, sustained hyperglycemia damages multiple organ systems, including renal, neuronal, and cardiovascular tissues, making DM a leading cause of morbidity and mortality worldwide.^2^^,^^3^

Among its subtypes, type 2 DM (T2DM) accounts for the vast majority of cases (> 95%) of DM and is often associated with obesity and insulin resistance.^1^^,^^2^ The progression of the disease is characterized by beta-cell demise, manifested through beta-cell death, dysfunction, and/or *trans*-/dedifferentiation.^4^^,^^5^^,^^6^^,^^7^ As beta-cell failure advances, glycemic control deteriorates and pharmacological interventions become necessary.^2^ Common drugs prescribed for T2DM, such as incretin mimetics, insulin, and sodium glucose cotransporter-2 inhibitors, primarily target obesity and blood glucose levels,^8^^,^^9^^,^^10^ but fail to halt the progressive loss of functional beta-cells that underlies disease progression.^8^^,^^9^^,^^10^^,^^11^^,^^12^ For these reasons, additional drugs are sought after to optimize and broaden treatment options.

A potential candidate for T2DM intervention is dextrorphan (DXO), the metabolite of the antitussive prodrug dextromethorphan (DXM),^9^^,^^13^^,^^14^ since several studies have highlighted the ability of DXO to reduce blood glucose concentrations in multiple mouse models of diabetes and in humans with T2DM,^9^^,^^15^^,^^16^^,^^17^^,^^18^ being part of placebo-controlled randomized clinical trials.^9^^,^^19^ Most notably, DXO protects mouse and human pancreatic islets from cell death induced by multiple stressors, including streptozotocin (STZ).^15^^,^^20^ This toxin enters beta-cells through glucose transporter-2 (GLUT2), causes deoxyribonucleic acid (DNA) damage by alkylation and oxidative stress, and is often used to study beta-cell death under diabetogenic conditions.^15^^,^^21^^,^^22^^,^^23^^,^^24^ Mechanistically, DXO induces activating transcription factor 4 (ATF4),^15^ which promotes serine-linked mitochondrial one-carbon metabolism (OCM) and increases the ratio of reduced to oxidized nicotinamide adenine dinucleotide phosphate (NADPH/NADP^+^) to counteract oxidative stress.^15^^,^^25^ The metabolic shift of glucose catabolism from the production of adenosine triphosphate (ATP) via the tricarboxylic acid (TCA) cycle to serine synthesis and NADPH via the serine-linked OCM has been proposed to promote beta-cell survival in the presence of STZ.^15^ Further, ATF4 has been shown to induce expression of the cytoprotective transcription factor nuclear factor erythroid 2-related factor 2 (*Nfe2l2* encoding NRF2), a central promoter of redox homeostasis.^26^^,^^27^^,^^28^^,^^29^ In contrast, ATF4 has also been shown to induce the cell death-promoting chaC glutathione-specific γ-glutamylcyclotransferase 1 (*Chac1* encoding CHAC1),^26^^,^^27^ which is also induced in beta-cells by cysteine deprivation to provide cells with cysteine from glutathione (GSH) degradation.^30^

In this study, we present the paradoxical observation that the islet cell-protective drug DXO upregulates CHAC1, which normally induces cell death in multiple different cell types.^31^^,^^32^^,^^33^ Further, CHAC1 is upregulated by erastin, an inducer of ferroptosis, in human pancreatic islets, correlating with their demise.^34^ By using a number of loss-of-function (LoF) and gain-of-function (GoF) experiments, we provide evidence that the anti-diabetic N-methyl-D-aspartate (NMDA) receptor antagonist DXO induces the pro-death CHAC1, which contributes to stabilizing the low protein levels of NRF2 in islets, resulting in a net islet cell-protective effect.

## Results

### Treatment of pancreatic islets with DXO induces CHAC1 that counteracts islet cell protection

**Figure 1:**
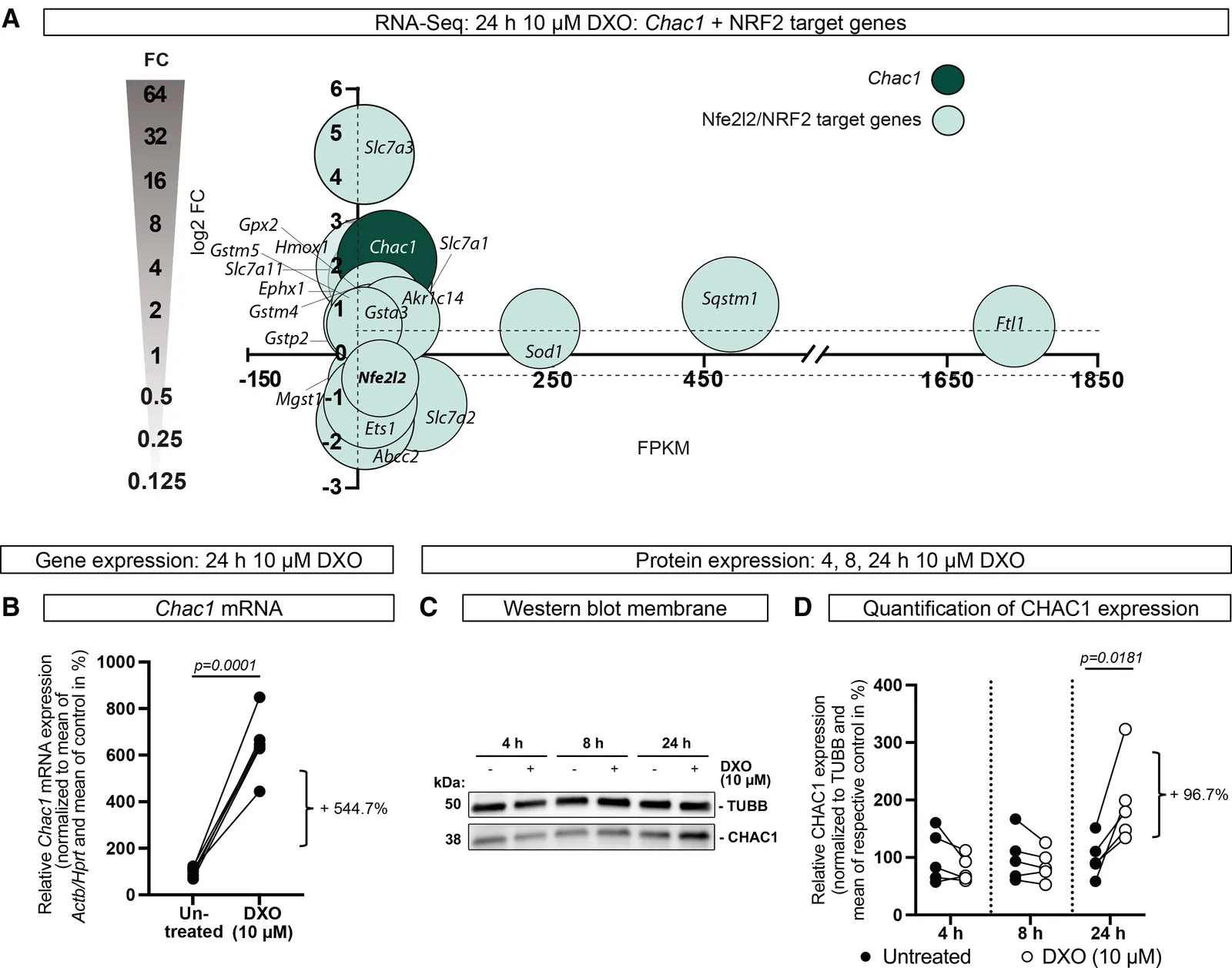
Induction of chaC glutathione-specific γ-glutamylcyclotransferase 1 in pancreatic islets by NMDA receptor antagonist dextrorphan (A) Transcriptomic analysis of *Chac1* and canonical nuclear factor erythroid 2-related factor 2 (NRF2, encoded by *Nfe2l2*) target genes. Gene expression is illustrated as log_2_ fold change (log_2_FC) relative to control, with thresholds set at ±0.5 log_2_FC. The plot displays log_2_FC (*y* axis) against transcript abundance fragments per kilobase of transcript per million mapped reads (FPKM, *x* axis); bubble size reflects 1-*p* values from RNA-seq (Pelligra et al., 2023). Horizontal dashed lines denote log_2_FC cutoffs; the gradient on the left indicates the magnitude of fold change (FC). (B) Relative *Chac1* mRNA expression following 24 h treatment of mouse pancreatic islets with 10 μM DXO. Expression was normalized to *Actb* and *Hprt* and the mean of the untreated controls. Data represent individual values (*n* = 6 independent experiments). Significant outliers (*p* value ≤ 0.05) identified by Grubbs’ test for outliers were excluded. (C) Immunoblot analysis of CHAC1 and TUBB (taken as a housekeeping protein) in islets treated ±10 μM DXO for 4, 8, or 24 h. (D) Corresponding quantification, normalized to TUBB and the mean of the 4 h untreated controls. Data represent individual values (*n* = 5 independent islet isolation). Image brightness and contrast in (C) were adjusted for better visualization. Statistical analyses: two-tailed paired Student’s *t* test for (B) and (D). Data in (A) were previously published in Pelligra et al. (2023).^15^

NRF2 is a key regulator of the cellular antioxidant response that controls the expression of genes involved in redox homeostasis and GSH metabolism.^35^ To determine whether antioxidant defense pathways were altered upon treatment of islets with the cytoprotective drug DXO, we examined the expression of established NRF2 target genes (Figure 1A) as well as CHAC1 expression, which promotes GSH degradation and thereby counteracts NRF2-driven GSH accumulation.^27^^,^^36^^,^^37^^,^^38^ The analysis was also motivated by the finding that DXO protects pancreatic islets from oxidative stress-induced cell death,^23^ and that DXO was found to reduce reactive oxygen species (ROS) in islets (Figure S1A). Previously generated bulk RNA-sequencing (RNA-seq) data were therefore used from mouse pancreatic islets treated with 10 μM DXO for 24 h, a condition that protects islets from cell death induced by STZ and other diabetogenic stressors, including inflammatory cytokines and H_2_O_2_.^9^^,^^15^^,^^23^ Differential gene expression analysis revealed that many of the previously reported NRF2 target genes were upregulated, such as solute carrier family 7 member 11 *(Slc7a11),* heme oxygenase 1 *(Hmox1),* and sequestosome 1 (*Sqstm1*) (Figure 1A). The genes were compiled from published studies using a curated set of canonical NRF2 target genes reported in NRF2 chromatin immunoprecipitation sequencing (ChIP-seq), knockdown experiments, and functional studies (Table S1),^39^^,^^40^^,^^41^^,^^42^ and only the genes that were differentially expressed were shown in our dataset. Unexpectedly, DXO increased *Chac1* mRNA expression while slightly decreasing the expression of *Nfe2l2*, the gene encoding NRF2 (Figure 1A). In addition, DXO, rather than the beta-cell toxin STZ, was found to upregulate the CHAC1 protein (Figure S1B). These observations were surprising given the reported pro-death role of CHAC1,^31^^,^^32^^,^^43^ and NRF2 being reported to promote cell survival and antioxidant defense, including in tissues such as pancreatic islets.^35^^,^^44^^,^^45^^,^^46^^,^^47^ To validate these counterintuitive observations, the upregulation of *Chac1* was reexamined by quantitative real-time PCR (RT-qPCR), and indeed, we detected a substantial increase of *Chac1* mRNA expression in islets after DXO treatment (Figure 1B). To assess CHAC1 protein levels over time, we treated islets with DXO for 4, 8, and 24 h. While no significant changes were observed at 4 and 8 h, a 2-fold increase in CHAC1 protein expression became evident after 24 h (Figures 1C and 1D). Notably, this time point coincided with the onset of DXO-mediated islet cell protection.^15^

**Figure 2:**
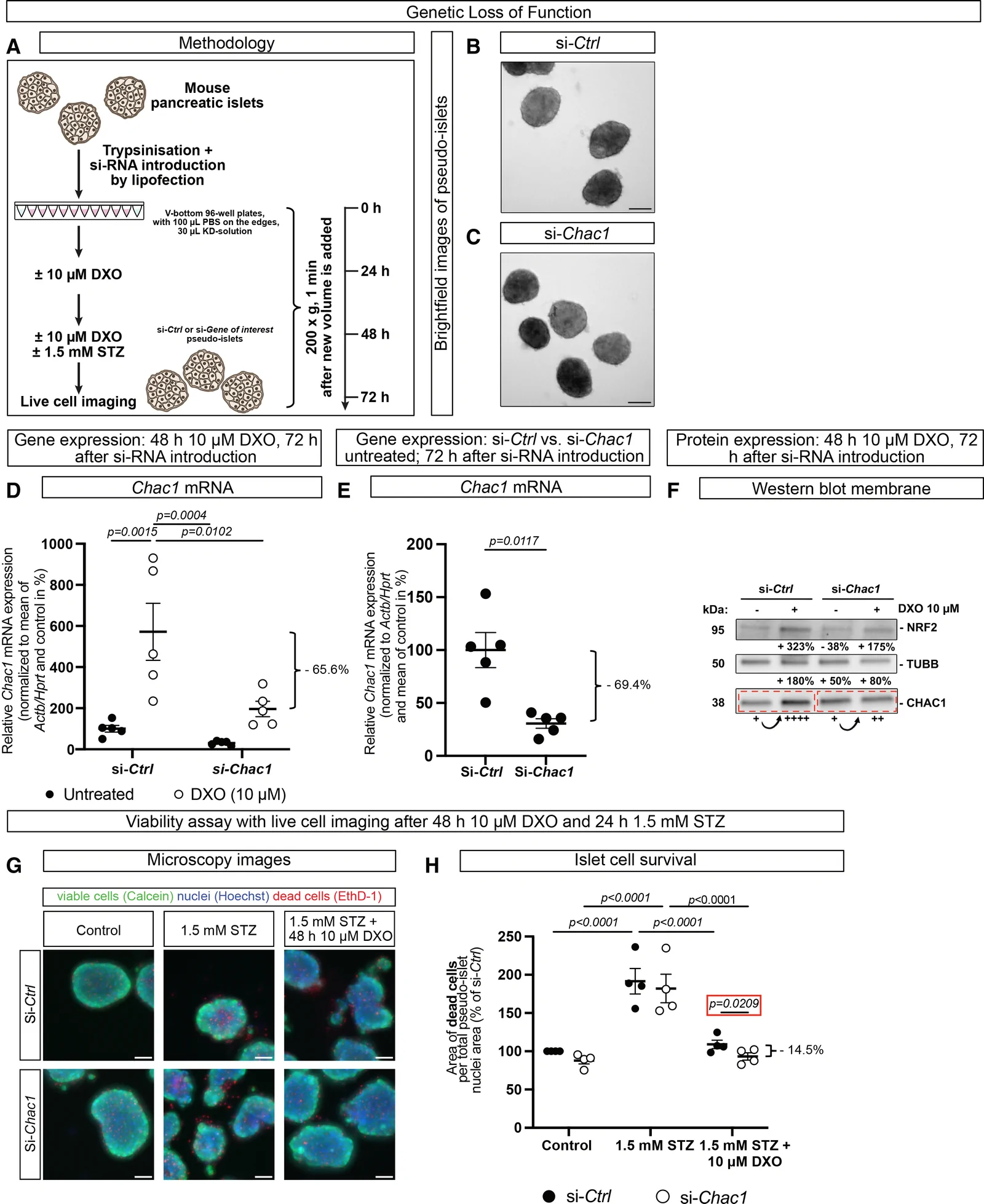
*Chac1* knockdown uncovers counteraction of CHAC1 to DXO-mediated islet cell protection (A) Schematic illustration of the method used for the generation of pseudo-islets silenced for *Chac1*. Dispersed mouse pancreatic islet cells are reaggregated with 50 nM siRNA in V- or U-bottom 96-well plates for 72 h. Every 24 h new medium with or without additions is added, followed by centrifugation at 200 × g for 1 min to ensure the collection of the cells at the bottom of the wells. (B and C) Representative images of si-*Ctrl* and si-*Chac1* treated mouse pseudo-islets, 72 h after 50 nM siRNA introduction. (D and E) (D) Relative mRNA expression of *Chac1* in pseudo-islets after treatment with 10 μM DXO (48 h) and si-*Ctrl* and si-*Chac1* siRNA (72 h), and (E) si-*Ctrl* vs. si-*Chac1* without DXO for better visualization of the data in (D). Data are shown as individual values normalized to the mean of housekeeping genes *Actb* and *Hprt* and the si-*Ctrl*; *n = 5* independent experiments. (F) NRF2/CHAC1/TUBB immunoblot of mouse pseudo-islets treated with 10 μM DXO (48 h) as well as si-*Ctrl* and si-*Chac1* siRNAs (72 h). Dashed red boxes highlight that DXO increased CHAC1 in *si-Ctrl*, but not in *si-Chac1* treated islets. Replicated in three of four independent experiments. The contrast and brightness of the western blot were adjusted for better visibility of the bands. (G) Images of live-dead imaging of si-*Ctrl* and si-*Chac1* treated pseudo-islets with or without 1.5 mM STZ (24 h) ± 10 μM DXO (48 h). The nuclei were stained with Hoechst, living cells with calcein and dead cells with ethidium homodimer-1 (EthD-1). (H) Quantification of cell death (*n = 4* experiments). Data are shown as individual values (mean ± SEM) with corresponding *p* values, and calculated as percentage of untreated control islets. Statistical significance was determined by repeated measures (RM) (H) or ordinary (D) two-way ANOVA with Tukey’s multiple comparisons, and a Welch’s t-test in (E). Scale bars, 100 μm for (B) and (C); 50 μm in (G).

Next, to investigate how DXO upregulates the pro-death CHAC1 protein, while maintaining islet cell protection, we silenced *Chac1* in dissociated mouse islet cells by using two different small interfering RNAs (siRNAs) (Figures 2 and S1C–S1E) that we reassociated to pseudo-islets using a previously described method.^48^ The pseudo-islets were generated to restore paracrine interactions between the islet cells,^49^ and their size and shape were similar to those of isolated mouse islets^50^ (Figures 2A–2C). In pseudo-islets, siRNA-mediated gene knockdown reduced the *Chac1* mRNA levels by 65%–70% or 80%–83%, depending on the siRNA used (Figures 2D, 2E and S1C). Since CHAC1 protein expression is low under control conditions, the higher CHAC1 protein levels induced by DXO allowed us to better visualize knockdown of the protein. Notably, DXO no longer upregulated CHAC1 when its RNA was silenced (compare red dashed boxes in Figure 2F).

We next asked whether reduced CHAC1 protein levels influenced islet cell viability in the presence of the beta-cell toxin STZ. To address this, live-dead imaging of siRNA-treated pseudo-islets was conducted with and without STZ treatment (Figures 2G, 2H, S1D and S1E), as previously described.^9^^,^^23^ While STZ efficiently induced cell death in islets, DXO provided almost complete protection (Figures 2H and S1E). However, gene knockdown of *Chac1* further enhanced DXO-mediated islet cell protection depending on the knockdown efficiency (Figures 2H and S1E). At a high knockdown efficiency, silencing *Chac1* even protected the pseudo-islets from STZ-induced cell death in the absence of DXO (Figure S1E). Previously, CHAC1 has been associated with cell death promoting pathways in other cell types,^36^^,^^43^ and since *Chac1* knockdown increased islet cell viability depending on the knockdown efficiency, the data suggest that CHAC1 partially counteracts the islet cell-protective effects of DXO.

### DXO-mediated stabilization of the NRF2 protein, but not of its mRNA

**Figure 3:**
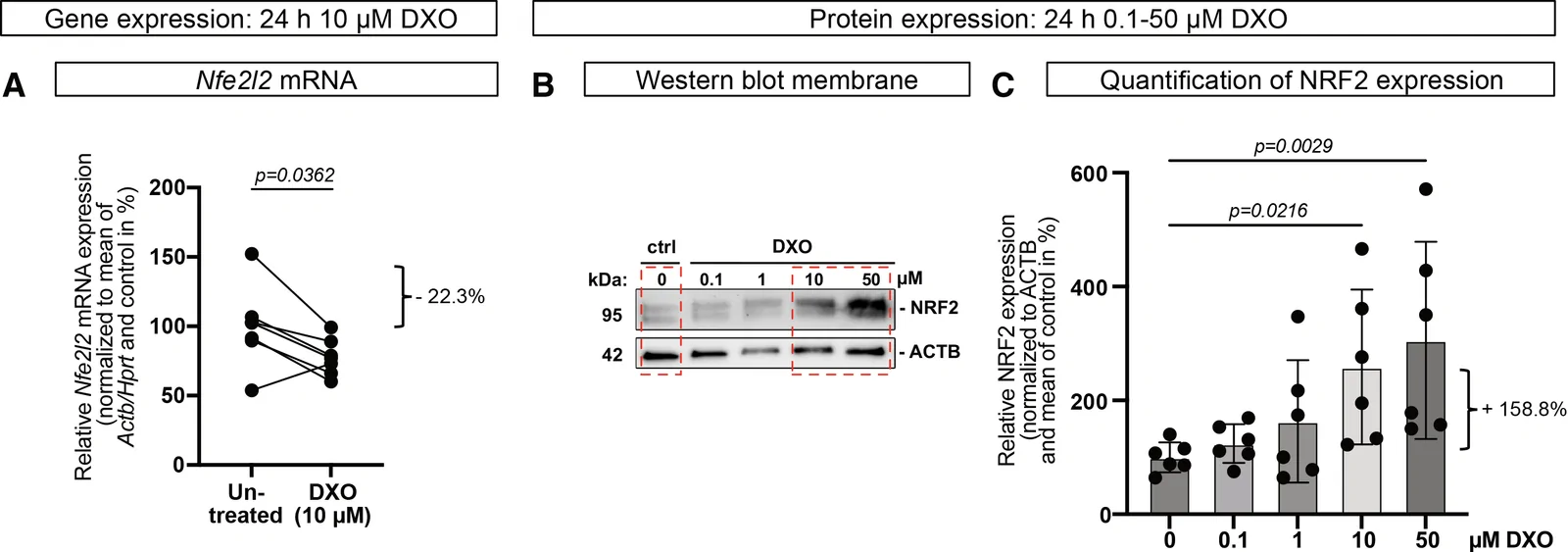
Dose-dependent upregulation of the NRF2 protein in pancreatic islets by dextrorphan (A) Relative mRNA expression of *Nfe2l2* (encoding NRF2) in mouse pancreatic islets after incubation with 10 μM DXO for 24 h. Values were normalized to the mean of housekeeping genes *Actb* and *Hprt* and the mean of the untreated control*. n = 7* independent islet isolations. (B) NRF2/ACTB immunoblot of mouse pancreatic islets treated with 0, 0.1, 1, 10, and 50 μM DXO for 24 h. (C) Quantification of NRF2 protein normalized to ACTB and the mean of the untreated controls. Data are shown as individual values (mean ± SEM); *n = 6* independent islet isolations. The contrast and brightness of (B) were adjusted for better visibility of the bands. For statistical evaluation a two-tailed paired Student’s *t* test was performed for (A) and a repeated measures (RM) one-way ANOVA with Dunnett’s multiple comparisons test for (C).

To investigate the discrepancy between the RNA-seq analysis that revealed a slight downregulation of the *Nfe2l2* transcript following DXO treatment (Figure 1A) and the observed increase in NRF2 protein expression after DXO treatment (Figure 2F), we examined whether the observed reduction of *Nfe2l2* mRNA could be independently validated. Consistent with the RNA-seq data, RT-qPCR confirmed a small but significant decrease in *Nfe2l2* mRNA expression in islets starting at 4 h after treatment with DXO (Figures 3A and S2A). In contrast, immunoblot analyses of pseudo-islets treated with DXO demonstrated a dose- and time-dependent upregulation of the NRF2 protein (Figures 3B and S2B). The upregulation of the NRF2 protein became statistically significant at a concentration of 10 μM DXO or higher (Figure 3C). To determine whether the increased NRF2 abundance was due to enhanced protein stability, we performed cycloheximide (CHX) chase assays for CHAC1 and NRF2, since CHX blocks protein synthesis.^51^ DXO was found to stabilize both proteins throughout the 4 h CHX treatment (Figure S2C), potentially by reducing their ubiquitination and degradation, as previously reported.^52^^,^^53^ The findings therefore indicate that DXO does not induce *Nfe2l2* at the mRNA level but instead increases NRF2 protein abundance by stabilizing the protein.

### NRF2 is required for full DXO-mediated islet protection from STZ-induced cell death

**Figure 4:**
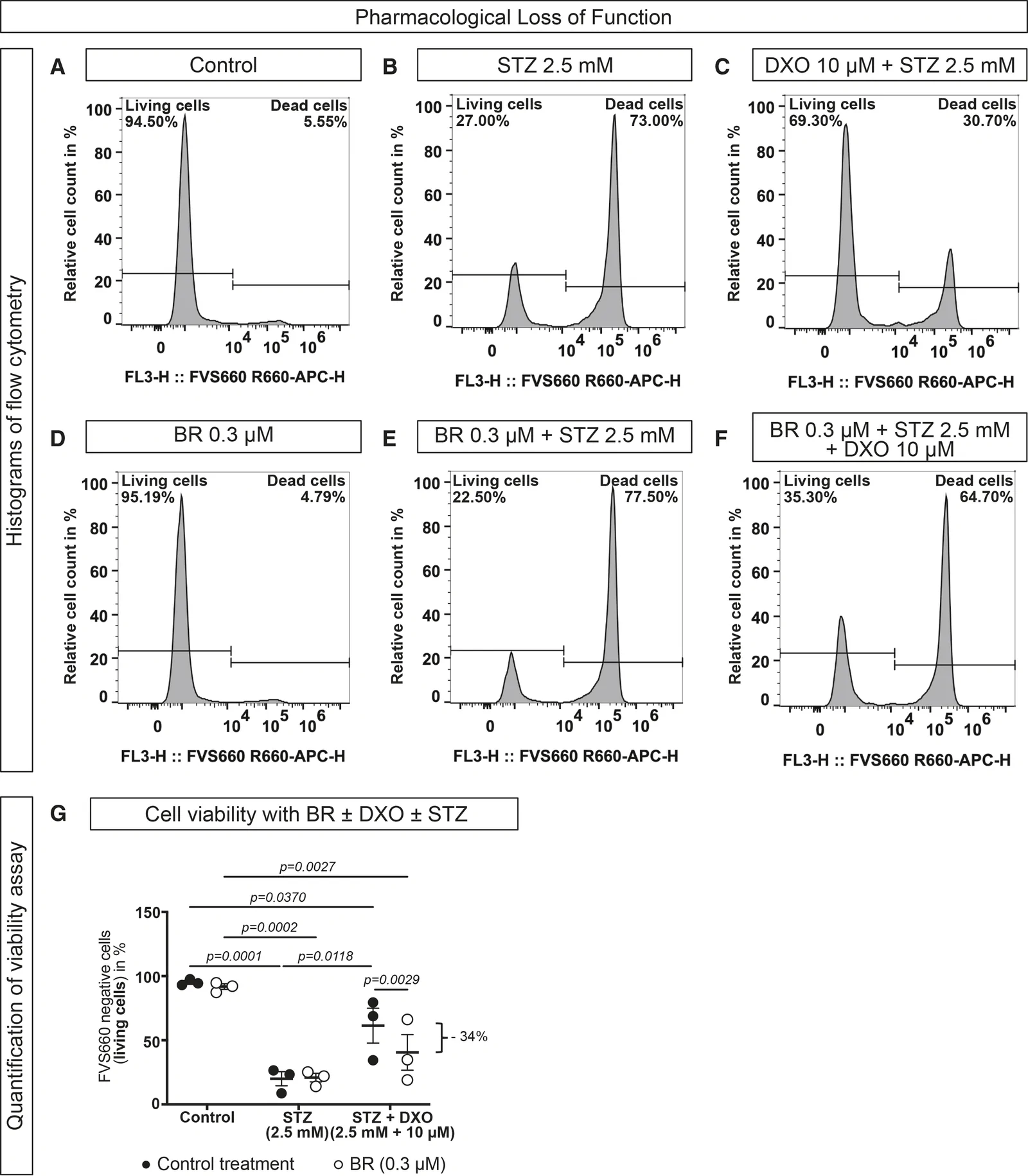
Treatment of pancreatic islets with the NRF2 inhibitor brusatol reveals a requirement of NRF2 for DXO-mediated islet cell protection (A–F) Representative histograms from a viability assay of untreated mouse pancreatic islets and islets treated with 2.5 mM STZ for 24 h, either alone or in the presence of 0.3 μM BR (4 h pretreatment + 4 h cotreatment) ± 10 μM DXO (48 h), including DMSO as vehicle control. (A) Control, (B) STZ, (C) STZ + DXO, (D) BR, (E) BR + STZ, and (F) BR + STZ + DXO. FVS660 negative populations represent living cells, while FVS660 positive populations indicate dead cells. Represented as relative cell count in %. (G) Quantification of the viability assay (*n* = 3 independent experiments). Data are presented as the percentage of living cells and shown as individual values (mean ± SEM) with corresponding *p* values. Statistical analysis was performed using RM two-way ANOVA with Tukey’s multiple comparisons post hoc test. Please note that Figures 4 and 6 share the same control groups for the analysis.

Next, we wished to determine the role of NRF2 in DXO-mediated islet cell protection using pharmacological and genetic LoF experiments (Figure 4). Therefore, we isolated primary mouse islets and pretreated them with DXO and brusatol (BR), an NRF2 inhibitor that promotes NRF2 degradation.^54^^,^^55^ To induce cell death, islets were treated with STZ (1) alone, (2) in combination with DXO, or (3) in combination with DXO plus BR (Figures 4 and S3). The islets were then dissociated and stained with the fixable viability stain FVS660 to determine cell death via flow cytometry. Consistent with our results from live-dead imaging (Figures 2G and 2H), STZ treatment markedly increased the proportion of dead cells, indicating substantial cytotoxicity (Figures 4A, 4B, and 4G).^15^^,^^20^ Moreover, treatment with DXO significantly protected pancreatic islets from STZ-induced cell death, as reflected by a substantial increase in viable and decrease in dead islet cells (Figures 4B, 4C, and 4G). Notably, when NRF2 was inhibited by BR, DXO no longer protected the islets efficiently from STZ-induced cell death (Figures 4D–4G).

**Figure 5:**
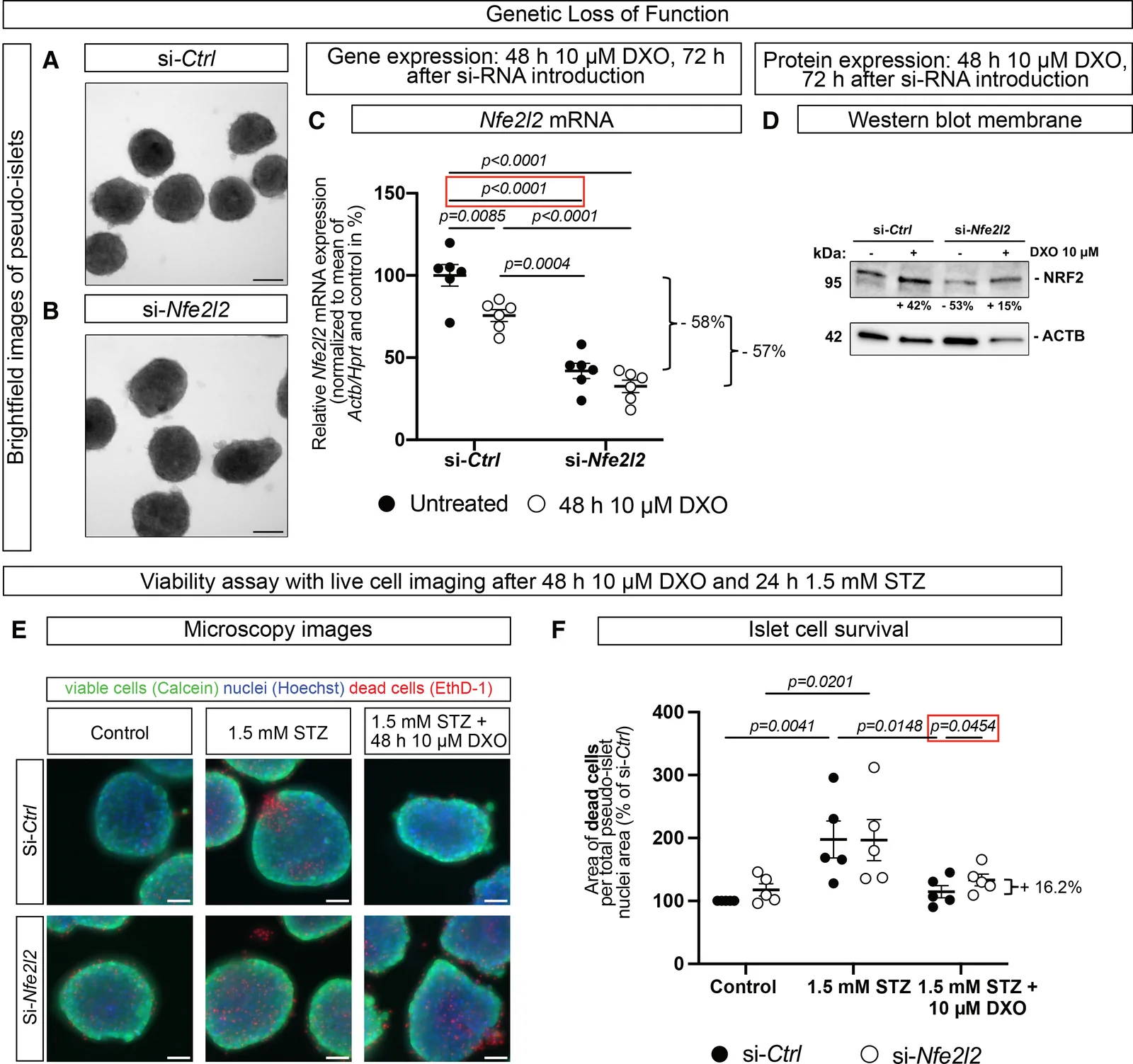
Knockdown of *Nfe2l2* reveals a role of NRF2 in DXO-mediated islet cell protection (A and B) Representative images of mouse pseudo-islets after 72 h treatment with 50 nM si-*Ctrl* and si-*Nfe2l2* siRNA. (C) Relative mRNA expression of *Nfe2l2* in pseudo-islets after treatment with 10 μM DXO (48 h) and 50 nM si-*Ctrl* or si-*Nfe2l2* siRNA (72 h). Values were normalized to the mean of housekeeping genes *Actb* and *Hprt* and the untreated si-*Ctrl.* Data are shown as individual values; *n = 6* independent experiments. (D) NRF2/ACTB immunoblot of mouse pseudo-islets treated with 10 μM DXO (48 h) as well as si-*Ctrl* and si-*Nfe2l2* siRNAs (72 h), reproduced with similar results in 4 out of 5 blots. The contrast and brightness of the western blot were adjusted for better visibility of the bands. (E) Images of live-dead staining of si-*Ctrl* and si-*Nfe2l2* treated pseudo-islets untreated or treated with 1.5 mM STZ (24 h) ± 10 μM DXO (48 h). The nuclei were stained with Hoechst, living cells with calcein and dead cells with ethidium homodimer-1 (EthD-1). (F) Quantification of cell death in untreated and STZ-treated mouse islets ±10 μM DXO for 48 h (*n = 5* experiments). Data are shown as individual values (mean ± SEM) with corresponding *p* values and calculated as percentage of untreated *si-Ctrl*. Statistical significance was determined by RM (F) or ordinary (C) two-way ANOVA with Tukey’s multiple comparisons test. Scale bars, 100 μm in (A) and (B); 50 μm in (E).

To validate the contribution of NRF2 to the protective effects of DXO (since BR can also reduce the protein levels of additional factors involved in cell survival^54^^,^^55^^,^^56^), we next depleted NRF2 from islet cells by siRNA-mediated knockdown of *Nfe2l2* (Figure 5). To this end, we used different siRNAs with different knockdown efficiencies (Figures 5C and S4). Similar to *Chac1* knockdown, morphological assessment of pseudo-islets did not reveal any obvious differences between islets treated with *Ctrl* siRNA and *Nfe2l2* siRNA (Figures 5A and 5B). Knockdown efficiency was determined by RT-qPCR, with *Nfe2l2* mRNA being reduced by around 58% or 80% in siRNA-treated pseudo-islets (Figures 5C and S4A). Immunoblot analyses also demonstrated a marked decrease in NRF2 protein abundance in the *Nfe2l2* siRNA-treated pseudo-islets (Figure 5D). In contrast, *Nfe2l2* silencing did not alter expression of *Chac1*/CHAC1 on mRNA and protein level (Figure S5).

Next, we wished to find out whether NRF2 was required for the cell protective effect of DXO. Therefore, pseudo-islets were incubated with DXO and STZ upon *Nfe2l2* silencing, and live-dead imaging was subsequently performed (Figures 5E and S4B). Analysis of live-dead imaging revealed that depletion of NRF2 attenuated the islet cell-protective effect of DXO (Figures 5F and S4C), with higher knockdown efficiencies more strongly inhibiting DXO-mediated cytoprotection (Figures S4B and S4C). In contrast, no effect of NRF2 depletion was observed in the absence of DXO (Figures 5F and S4C), irrespective of the knockdown efficacy. The data demonstrate that NRF2 is essential for DXO to fully protect pancreatic islets against STZ-induced cell death.

### Pharmacological activation of NRF2 protects islet cells without enhancing DXO-mediated cytoprotection

**Figure 6:**
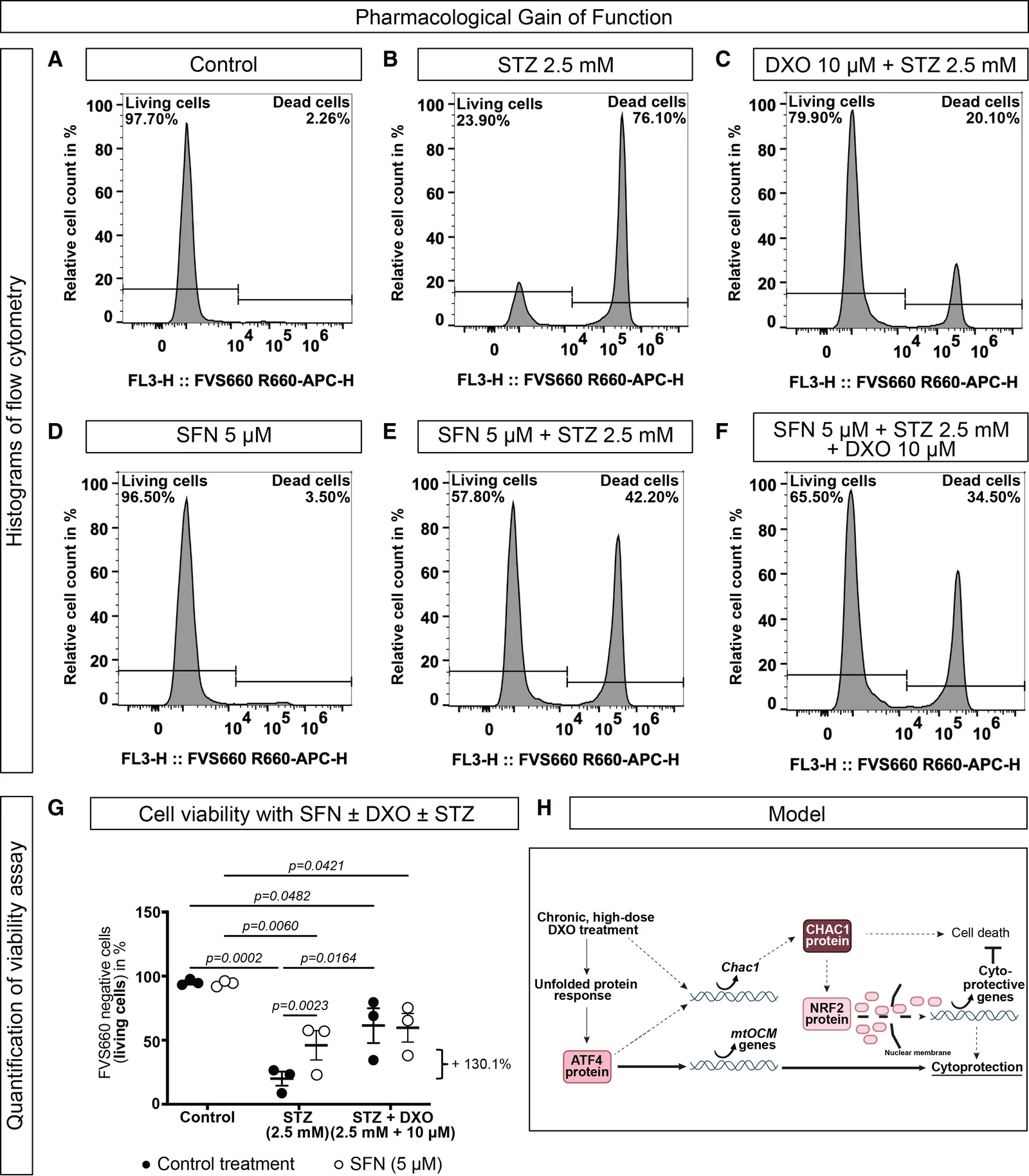
Activation of NRF2 by sulforaphane reveals role of NRF2 in islet cell protection from STZ-mediated cell death (A–F) Representative histograms from a viability assay of untreated mouse pancreatic islets and islets treated with 2.5 mM streptozotocin (STZ) for 24 h, either alone or in the presence of 10 μM DXO (48 h) or ± 5 μM SFN (48 h), including dimethyl sulfoxide (DMSO) as vehicle control. (A) Control, (B) STZ, (C) STZ + DXO, (D) SFN, (E) SFN + STZ, and (F) SFN + STZ + DXO. Fixable viability stain 660 (FVS660) negative populations represent living cells, while FVS660 positive populations indicate dead cells. Represented as relative cell count in %. (G) Quantification of the cell viability assays (*n = 3* independent experiments). Data are presented as the percentage of living cells and shown as individual values (mean ± SEM) with corresponding *p* values. (H) Model derived from our data. As reported previously by (Pelligra et al., 2023), DXO increases expression of ATF4 that activates serine-linked mitochondrial one-carbon metabolism (mtOCM) genes in pancreatic islets. Based on the data shown here, we propose that DXO treatment induces *Chac1*/CHAC1 expression and stabilizes NRF2 protein concentrations in islet cells. Even though CHAC1 induces cell death, the robust increase in NRF2 protein induced by DXO results in a net islet cell protective effect of DXO. Statistical analysis was performed using RM two-way ANOVA with Tukey’s multiple comparisons post hoc test. Figures 4 and 6 share the same control groups.

To test if activating NRF2 protects islets from cell death, we performed pharmacological GoF experiments (Figure 6). More specifically, mouse pancreatic islets were treated with DXO and sulforaphane (SFN), a well-characterized NRF2 activator that promotes NRF2 stabilization by altering the Kelch-like ECH-associated protein 1 (KEAP1)-NRF2 protein complex.^57^^,^^58^^,^^59^ SFN strongly upregulated NRF2 expression in the islets (Figure S6). Treatment with either DXO or SFN was found to independently protect islets from STZ-induced cell death (Figures 6A–6G). However, SFN did not increase the cell protective effect of DXO (Figure 6G). These results show that NRF2 activation is sufficient to promote islet survival and that DXO-mediated cytoprotection cannot be further enhanced by SFN.

Our findings indicate a mechanism of action of DXO that involves stabilization of NRF2, which is required and sufficient to protect islets from STZ-mediated cell death (Figure 6H).

## Discussion

Beta-cell demise is a major determinant of T2DM progression, leading to elevated blood glucose concentrations, intensified drug treatment, and an increased risk of diabetic long-term complications.^60^ However, there is currently no marketed therapy that directly targets and effectively halts or reverses beta-cell loss.^5^^,^^12^ In recent years, the antitussive prodrug DXM has gained attention for its islet cell-protective properties and its promoting effect on glucose-stimulated insulin secretion (GSIS).^15^^,^^61^ However, the molecular mechanisms of action underlying the cytoprotective effects of the NMDA receptor antagonist DXO, the main metabolite of DXM, are only starting to be uncovered.

Here, we demonstrate that treatment of mouse islets with DXO paradoxically induces, at the mRNA and protein levels, a substantial upregulation of *Chac1*/CHAC1, which is known to deplete cells of GSH and induce cell death in multiple cell types.^27^^,^^31^^,^^32^^,^^36^^,^^43^^,^^62^ Further, its upregulation correlates with islet cell demise in mice and humans.^30^^,^^34^ STZ is a diabetogenic beta-cell toxin that is widely used to induce islet cell death and diabetes by inducing DNA damage and oxidative stress in beta-cells, events described in human individuals with diabetes.^6^^,^^21^^,^^22^^,^^63^^,^^64^ In our study, strong depletion of CHAC1 in islet cells did not reduce basal cell death, but decreased STZ-induced cell death. Furthermore, CHAC1 depletion enhanced DXO-mediated protection from STZ toxicity, consistent with a pro-death function of CHAC1.^31^^,^^32^^,^^34^^,^^36^^,^^37^^,^^43^

Abundance of *Nfe2l2* mRNA was, again counterintuitively, mildly downregulated by DXO. However, we could show that its protein levels increased with increasing DXO concentrations and that the NRF2 protein got stabilized by DXO, consistent with lower ROS levels in DXO-treated islets. The inverse correlation between the mRNA and protein expression of NRF2 that we report for pancreatic islets, is consistent with previous studies on immune cells, yeast, and several cell lines.^65^^,^^66^^,^^67^ In addition, pharmacological and genetic activation of NRF2 in rodents and human cell lines have been shown to decrease inflammation, enhance insulin sensitivity and secretion, reduce body weight, preserve beta-cell mass, and even prevent the onset of T2DM in mice.^44^^,^^45^^,^^46^^,^^47^^,^^68^^,^^69^ Through multiple LoF and GoF experiments, we demonstrate that NRF2 is required for full DXO-mediated islet protection and is sufficient to shield islets from STZ-induced cell death. This aligns with previous studies showing a protective role of NRF2 in islets.^45^^,^^70^^,^^71^ Furthermore, the upregulation of multiple NRF2 target genes by DXO supports the conclusion that DXO stabilizes NRF2 to protect islet cells from death.

In summary, we show that the islet-protective agent DXO, the active metabolite of the antidiabetic and antitussive NMDA receptor antagonist DXM, induces both NRF2 and CHAC1 proteins. While NRF2 is required for DXO-mediated protection, CHAC1 acts to reduce it. The findings indicate that this therapeutic agent, which preserves beta-cell mass and prevents cell death in human islets, mouse islets, and multiple diabetic models (including *db/db* and *NOD*), coordinates its protective effects through a sophisticated mechanism of action involving counteracting components.

### Limitations of the study

This study has several limitations. First, INS-1E cells represent a reliable and useful rat cell line to study multiple aspects of beta-cell physiology.^72^^,^^73^ However, even though these cells responded to genetic and pharmacologic NMDA receptor inhibition by increasing GSIS,^9^ they did not exhibit a measurable cell protection by DXO (data not shown). This circumstance prevented the establishment of complementary *in vitro* cell protection experiments using this insulinoma cell line. Second, the amount of primary tissue obtained during islet isolation was limited, restricting the range of downstream analyses that could be performed. In particular, assays requiring relatively large sample quantities, such as GSH measurements using commercial kits, could not be conducted. Finally, knockdown experiments in combination with pseudo-islet formation could not be conducted with human islet cells, since the latter only weakly aggregated to allow straightforward live-dead imaging.

## Resource availability

### Lead contact

Further information and request for resources and reagents should be directed to and will be fulfilled by the lead contact, Dr. Eckhard Lammert (lammert@hhu.de).

### Materials availability

This study did not generate new unique reagents.

### Data and code availability

RNA-sequencing datasets used in this study are available through the Mendeley Data repository under “Pelligra et al., 2023”. The corresponding DOI is provided in the key resources table. Unprocessed western blot images and flow cytometry analyses are included in the appendix. Additional datasets generated during the study, including microscopy and qPCR data, are available from the lead contact upon reasonable request. The code used for pancreatic islet analysis is identical to that described by Scholz et al. and Pelligra et al.^15^^,^^23^ and has been deposited in Zenodo; the DOI is listed in the key resources table. Any further information required to reproduce or reanalyze the data reported in this study can be obtained from the lead contact upon request.

## Acknowledgments

This work was supported and funded by the 10.13039/501100003484Heinrich Heine University Düsseldorf, the 10.13039/100031584German Center for Diabetes Research (DZD e.V.), Deutsche Forschungsgemeinschaft (DFG): RTG 2576 (vivid – *in vivo* investigations toward the early development of type 2 diabetes) and CRC 1774 (cardio-diabetes-crosstalk, A02) as well as the 10.13039/501100003107Federal Ministry of Health, and the 10.13039/501100014690Ministry of Culture and Science of North Rhine-Westphalia. We would like to acknowledge S. Jakob for technical support. We thank M. Müller, E. Huβ, R. Diaz, B.-F. Belgardt, A. Hamacher, and A. Guzman Carrasco for their fruitful discussions and input.

## Author contributions

C.W. designed, performed, analyzed, and interpreted most of the experiments with E.L.; P.K. helped with flow cytometry, western blots, analysis, and study design. B.B. and A.K. aided in live-dead imaging, RT-qPCR, and islet isolation. D.E. provided scientific and experimental advice and performed controls of data integrity and analyses. E.L. supervised and scientifically guided C.W. and P.K. throughout the study. E.L. and C.W. wrote the manuscript, while C.W. prepared the figures. All authors read and revised the manuscript.

## Declaration of interests

E.L. declares the following competing financial interests: the author is an inventor of the PCT application PCT/EP2023/058373 entitled “Synthesis of morphinans with reduced hERG and MOR binding,” US patent 10,464,904 entitled “Dextrorphan-derivatives with suppressed central nervous activity,” and the US patent 9,370,511 entitled “Morphinan-derivatives for treating diabetes and related disorders.”

## Declaration of generative AI and AI-assisted technologies in the writing process

During the preparation of this work the authors used OpenAI (2023) ChatGPT (GPT-4) and Google Gemini to improve language and readability of the text. After using these tools, the authors reviewed and edited the content as needed and took full responsibility for the content of the published article.

## STAR★Methods

### Key resources table

**Table:** 

REAGENT or RESOURCE	SOURCE	IDENTIFIER
**Antibodies**		

See Table S2	This paper; See Table S2	See Table S2

**Chemicals**		

Dextrorphan tartrate (DXO)	Sigma-Aldrich	Cat#D127
Brusatol	Sigma-Aldrich	Cat#SML1868
Sulphoraphane	BLDpharm	Cat#BD01371706
Lipofectamine RNAiMAX	Invitrogen by Thermo Fisher Scientific	Cat#13778–150
Streptozotocin	Sigma-Aldrich	Cat#S0130
Cycloheximide	Thermo Scientific Chemicals by Thermo Fisher Scientific	Cat#J66004.XF
Hydrogen peroxide 30%	Fischer Scientific by Thermo Fisher Scientific	Cat#10687022
Pluronic ^TM^ F-127 (20% in DMSO)	Invitrogen by Thermo Fisher Scientific	Cat#P3000MP

**Critical commercial assays**		

LUNA® Universal qPCR Master Mix	NEB	Cat#M3003E
SuperScript^TM^ II reverse transcriptase Kit	Invitrogen by Thermo Fisher Scientific	Cat#18064071
High-Capacity cDNA Reverse Transcription Kit	Invitrogen by Thermo Fisher Scientific	Cat#4368813
RNase-Free DNase Set	Qiagen	Cat#79254
RNeasy Mini Kit	Qiagen	Cat#74104

**Deposited data**		

RNA-sequencing raw data	Mendeley Data	https://doi.org/10.17632/g4bdvw6czr.2

**Experimental models: Organisms/strains**		

Mouse: C57BL/6J	Janvier Labs	RRID:IMSR_JAX:000664

**Oligonucleotides**		

See Table S3	This paper, See Table S3	See Table S3

**Software and algorithms**		

Code for pancreatic islet analyses of cell viability	Scholz et al.^23^, Pelligta et al.^15^; Zenodo	https://doi.org/10.5281/zenodo.5820007
Fiji (ImageJ) image analysis software	Schindelin et al.^74^	RRID: SCR_002285
FlowJo V10	BD Biosciences	RRID: SCR_008520
GraphPad Prism	GraphPad Software	RRID: SCR_002798
ImageLab	BioRad	RRID: SCR_014210
QuantStudio^TM^ Design and Analysis Desktop Software v1.5.1 QuantStudio^TM^ Design and Analysis Desktop Software v1.7.2	Applied Biosystems	N/A N/A

**Other**		

Fixable viability stain 600	BD Biosciences	Cat#564405
Hoechst 33342	Invitrogen by Thermo Fisher Scientific	Cat#H3570
LIVE-DEAD viability-cytotoxicity Kit	Invitrogen by Thermo Fisher Scientific	Cat#L3224
CellROX™ Orange	Invitrogen by Thermo Fisher Scientific	Cat#C10443
CM-H_2_DCFDA Kit with probenecid	Invitrogen by Thermo Fisher Scientific	Cat#C6827

### Experimental model and study participant details

#### Mouse models

For this study male C57BL/6J (Janvier) mice aged 9–12 weeks were sacrificed for their pancreatic islets. The mice were kept at the Zentrale Einrichtung für Tierforschung und wissenschaftliche Tierschutzaufgaben (ZETT) animal facility of the Heinrich-Heine-University (HHU) Düsseldorf, under conditions of 22°C, 55% humidity, and a 12 h light - 12 h dark day-night-cycle. They were provided standard laboratory feed and water *ad libitum*. Euthanasia was performed with carbon dioxide (CO_2_) exposure and cervical dislocation. All organs were obtained in accordance with German animal welfare regulations (internal ZETT number O1016/25) and with approval from the competent authority, the Landesamt für Verbraucherschutz und Ernährung Nordrhein-Westfalen (LAVE, North Rhine-Westphalia, Germany). As only young male mice (9–12 weeks of age) were included, sex- and age-specific effects could not be assessed, and the findings may not be generalizable to female or younger/older mice.

### Method details

Please note that some of the methods described here have already been described by Pelligra et al. (2023).^15^

#### Isolation of mouse pancreatic islets

Pancreatic islets were isolated from mice using a protocol adapted from Yesil et al. (2009)^75^ and was previously described in Pelligra et al. (2023).^15^ The pancreas was perfused via the bile duct with 3 mL Dulbecco’s Modified Eagle Medium (DMEM + GlutaMAX, 1 g/L glucose) (Gibco® by Life Technologies) containing 0.73% Liberase TL Research Grade (Roche). The pancreas was excised and incubated at 37 °C for 16.5 min for enzymatic digestion. Digestion was stopped by addition of DMEM supplemented with 15% heat-inactivated fetal bovine serum (FBS) (Gibco® by Life Technologies) and penicillin–streptomycin (Gibco® by Life Technologies). Samples were dissociated by vigorous shaking, filtered through a 40 μm mesh sieve, and centrifuged at 900 rpm for 3 min. Islets were purified by density gradient centrifugation using Lymphoprep (STEMCELL Technologies) (1,200 rpm, 25 min, no brake). The islet-containing interphase was collected, washed with DMEM containing 15% FBS (Gibco® by Life Technologies), and transferred to culture dishes. Islets were cultured in Connaught Medical Research Laboratories (CMRL) 1066 medium (Gibco® by Life Technologies) supplemented with 15% heat-inactivated FBS (Gibco® by Life Technologies), 100 U/mL penicillin (Gibco® by Life Technologies), 100 μg/mL streptomycin (Gibco® by Life Technologies), 50 μM beta-mercaptoethanol (Gibco® by Life Technologies), 0.15% sodium bicarbonate (Gibco® by Life Technologies), and 10 mM glucose (Sigma-Aldrich). Prior to experiments, islets were handpicked, washed, and cultured overnight at 37 °C in a humidified incubator with 5% CO_2_ in islet medium.

#### RNA-isolation

Total RNA was isolated from pancreatic islets using the RNeasy Mini Kit (Qiagen) according to the manufacturer’s instructions, including on-column DNase digestion (RNase-Free DNase Set, Qiagen). 10–40 islets were collected in 1 mL phosphate-buffered saline (PBS), pelleted by centrifugation (1 min, 900 × g), and lysed in 350 μL RLT buffer (Qiagen). Lysates were disrupted using a cell disruptor for 10 min at 4 °C and, when necessary, stored at −80 °C until further processing. After addition of 70% ethanol, samples were applied to RNeasy spin columns (Qiagen). Columns were washed with RW1 and RPE buffers (Qiagen), including on-column DNase digestion, and RNA was eluted in 30 μL RNase-free ddH_2_O (Qiagen).

#### Reverse transcriptase PCR and quantitative real-time PCR

The RT-qPCR was performed following the general approach described by Pelligra et al. (2023)^15^ with slight modifications. cDNA was synthesized from isolated RNA using the High-Capacity cDNA Reverse Transcription Kit (Thermo Fisher Scientific) according to the manufacturer’s instructions. Quantitative PCR (qPCR) was performed using LUNA® Universal RT-qPCR Reagent (New England Biolabs). Prior to amplification, cDNA was diluted 1:3 in nuclease-free ddH_2_O (Qiagen). Primer sequences used in this study are listed in Table S2. Reactions were run using either the QuantStudio™ 1 System (Applied Biosystems) or the QuantStudio™ 7 Flex Real-Time PCR System (Applied Biosystems) and analyzed using the QuantStudio™ Design and Analysis Desktop Software (Applied Biosystems).

#### Western blot

Western blots were performed similar to the methodology described by Pelligra et al. (2023)^15^ with modifications. Proteins were isolated from pancreatic islets by collecting 40–100 islets and washing them with ice-cold PBS followed by centrifugation (900 × g, 3 min). Pellets were lysed in 45 μL RIPA buffer supplemented with protease inhibitor cocktail (Roche) and disrupted for 10 min at 4 °C using a cell disruptor. Lysates were cleared by centrifugation (10,000 × g, 10 min, 4 °C), and the supernatant was collected. Samples were mixed with 4× Laemmli buffer (Bio-Rad) containing 40 mM NaF (Sigma-Aldrich) and 4% beta-mercaptoethanol (Roth) and denatured for 10 min at 95 °C (Analytik Jena). Proteins were separated on 4–15% gradient gels (Mini-PROTEAN® TGX Stain-Free Gels, Bio-Rad) alongside Precision Plus Protein Dual Color Standard (Bio-Rad) using Tris/Glycine/SDS running buffer (Bio-Rad) at 140 V for 60 min. Stain-Free activation and imaging were performed using the ChemiDoc MP Imaging System (Bio-Rad). Proteins were transferred to PVDF membranes using the Trans-Blot Turbo Mini 0.2 μm PVDF Transfer Pack (Bio-Rad) with the Trans-Blot Turbo transfer system (Bio-Rad). Membranes were blocked in 5% non-fat dried milk powder (AppliChem) in PBS-Tween 0.5% (Roth) for 1 h at room temperature and incubated with primary antibodies diluted in blocking solution (Table S3) for 1.5 h at room temperature or overnight at 4 °C. Following three washes with PBS-Tween 0.5% (Roth), membranes were incubated with secondary antibodies (Table S3) for 1 h at room temperature. After washing, signals were detected using Clarity Western ECL Substrate (Bio-Rad) or SuperSignal West Atto Ultimate Sensitivity Substrate (Thermo Fisher Scientific), and images were acquired using chemiluminescent and colorimetric detection on the ChemiDoc MP Imaging System (Bio-Rad). Band intensities were quantified using Image Lab Software (Bio-Rad, version 6.1). Protein levels were normalized to ACTB or TUBB and then normalized to the mean of the control group.

#### Pseudo-islet formation with siRNA transfection

Islets were collected and washed with pre-warmed Dulbecco’s PBS (Gibco® by Life Technologies), pelleted by centrifugation (900 rpm, 3 min), and dissociated using 0.05% Trypsin-EDTA (Gibco® by Life Technologies). Trypsinization was stopped with islet medium, and cells were pelleted and resuspended in islet medium. Cell numbers were determined by Trypan Blue using a Fast-read® counting chamber (Biosigma srl). Cells were transfected with control siRNA (*si-Ctrl*) or *Nfe2l2*-or *Chac1*-targeting siRNA (Dharmacon/Thermo Fischer Scientific) using Lipofectamine RNAiMAX Transfection Reagent (Thermo Fisher Scientific) in OptiMEM (Gibco® by Life Technologies). siRNA–lipid complexes were incubated for 5 min at room temperature and added to the cell suspension at a final siRNA concentration of 50 nM for Dharmacon and 100 nM for Thermo Fischer Scientific and a density of 1500 cells per 30 μL. For pseudo-islet formation, transfected cells were seeded into U- or V-bottom 96-well plates and centrifuged (200 × g, 1 min) to promote aggregation. Pseudo-islets were cultured for 1 day at 37 °C in a humidified incubator (5% CO_2_, 95% air) before treatment. After each treatment plates were centrifuged (200 × g, 1 min).

#### Measurement of islet cell viability in mouse pseudo-islets via microscopy

Cell viability in pseudo-islets after exposure to 1.5 mM STZ for 24 h was evaluated using the LIVE/DEAD Viability/Cytotoxicity Kit (Thermo Fisher Scientific), following the general approach described by Pelligra et al. (2023)^15^ with slight modifications. After treatment, pseudo-islets were incubated for 1 h at 37 °C (5% CO_2_) in a KRH buffer with 0.1% BSA (Sigma-Aldrich) and 10 mM glucose (Sigma-Aldrich) containing 10 μg/mL Hoechst 33342, 2 μM Calcein AM, and 4 μM Ethidium homodimer-1 (EthD-1) in the dark. z stack images were acquired using a Zeiss ApoTome (Carl Zeiss MicroImaging GmbH) with a Plan-Apochromat 10×/0.45 objective. Images were analyzed using Fiji (ImageJ),^74^ and cell death was quantified using a previously published semi-automated analysis macro.^15^^,^^23^ The EthD-1–positive area was normalized to the total Hoechst-positive islet area.

#### Measurement of islet cell viability via flow cytometry

Flow cytometry was performed following the methodology described by Pelligra et al. (2023)^15^ with slight modifications. 100 islets per condition were pretreated with the indicated compounds for up to 24 h and co-treated with 2.5 mM streptozotocin (STZ) for an additional 24 h. Islets were collected with media and washed with PBS (Gibco® by Life Technologies). Cells were trypsinized with 0.05% trypsin (Gibco® by Life Technologies), and the reaction was stopped with DMEM containing 10% FBS. A heat-treated sample (60 °C, 5 min) was included as a dead control. Cells were stained with FVS660 (1:1.000 in PBS; BD Biosciences) for 15 min at room temperature in the dark, washed twice with PBS, and resuspended in 300 μL PBS. Cell viability was assessed using a CytoFlex S flow cytometer (Beckman Coulter) at the DDZ. Data were analyzed in FlowJo (v10.10.00, BD Biosciences) with gating for singlets and live/dead populations.

#### Measurement of reactive oxygen species in mouse islets via microscopy

Cellular ROS levels in mouse islets following 24 h exposure to 10 μM DXO or 25 μM hydrogen peroxide (positive control) were assessed using CellROX Orange or CM-H_2_DCFDA (DCF) (Thermo Fisher Scientific), following the general approach described by the provider. After treatment, islets were incubated for 30 min at 37 °C (5% CO_2_) in a KRH buffer with 0.1% BSA (Sigma-Aldrich) and 10 mM glucose (Sigma-Aldrich), containing 10 μg/mL Hoechst 33342, and either 5 μM CellROX or 10 μM DCF with 20% pluronic F-127 (Thermo Fisher Scientific) 1:1 v/v and 1 mM probenecid (Thermo Fisher Scientific) in the dark. z stack images were acquired using a Zeiss ApoTome (Carl Zeiss MicroImaging GmbH) with a Plan-Apochromat 20×/10×/0.45 objective. Images were analyzed using Fiji (ImageJ),^74^ and mean intensity of the islets was measured.

### Quantification and statistical analysis

Statistical analyses were performed using GraphPad Prism 10.6.1. The specific tests applied are indicated in the Figure legends. Comparisons between two groups were made using Welch’s t-test or paired two-tailed Student’s t-tests, while multiple-group comparisons were conducted using (RM, repeated measures) one- or two-way ANOVA followed by Dunnett’s or Tukey’s post hoc tests. Data are presented as mean ± SEM unless otherwise noted. Microscopy-based cell viability analyses were performed under blinded conditions. For qPCR experiments significant outliers (*p* ≤ 0.05) identified by Grubb’s test were excluded. Experiments were not evaluated if there was <25% cell death observed in the STZ condition. The number of biological replicates and independent experiments is reported in the Figure legends.
