# Supplementary material for: CHAC1-linked NRF2 protein expression participates in pancreatic islet protection by dextrorphan
Source: iScience. 2026 Sep 7;29(9):117474. doi: 10.1016/j.isci.2026.117474 (PMC13578463; doi:10.1016/j.isci.2026.117474)
Supplement: Supplementary file 1 [file mmc1.pdf]

iScience, Volume *29*

Supplemental information

CHAC1-linked NRF2 protein expression participates in pancreatic islet protection by dextrorphan

Celine Weippert, Philip Kirschner, Barbara Bartosinska, Andrea Köster, Daniel Eberhard, and Eckhard Lammert

SUPPLEMENTAL INFORMATION

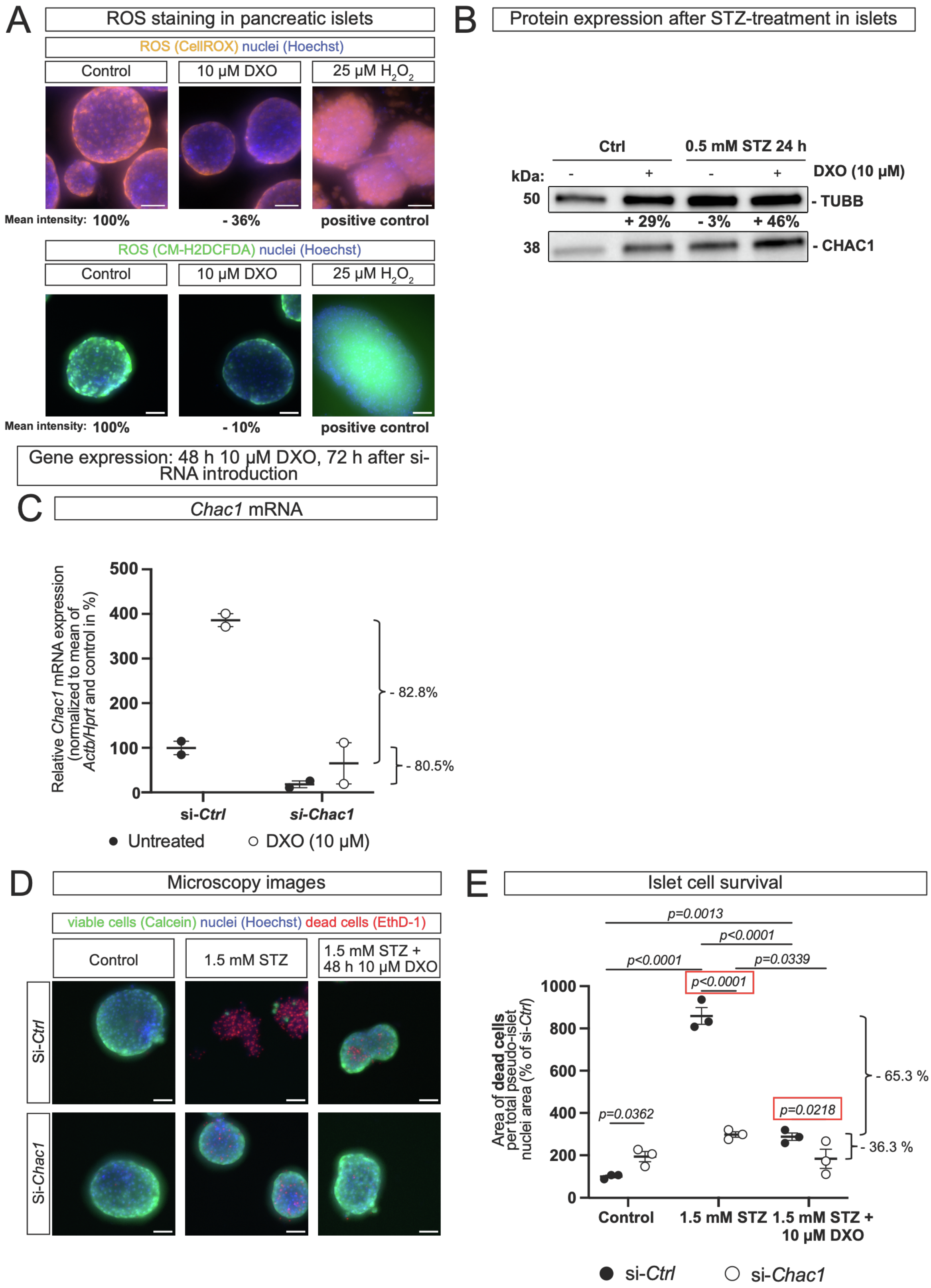

Figure S1. ROS reduction by DXO and enhanced islet cell protection by enhanced *Chac1* knockdown (related to Fig. 2). A, Staining with CellROX and CM-H_2DCFDA for cytoplasmic ROS and Hoechst for nuclei. Pancreatic islets were treated for 24 h ± 10 µM DXO and 25 µM H_2O_2 as a positive control. Representative images are from one independent islet isolation each. CellROX staining was performed on islets pooled from 4 mice, and CM-H_2DCFDA staining was performed on islets pooled from 6 mice. Approximately 6–11 islets per condition (control and DXO) were analysed. **B**, CHAC1/TUBB immunoblot of mouse pancreatic islets treated with 10 µM DXO for 48 h and ± 0.5 mM STZ for 24 h. The contrast and brightness of the western blot were adjusted for better visibility of the bands. *n = 1.* **C**, Relative mRNA expression of *Chac1* in pseudo-islets after treatment with 10 µM DXO (48 h) and si-*Ctrl* and si-*Chac1* siRNA (72 h, 100 nM). Data are shown as individual values normalized to the mean of housekeeping genes *Actb* and *Hprt* and the si-*Ctrl; n = 2.* **D**, Representative images of live-dead staining of si-*Ctrl* and si-*Chac1* treated pseudo-islets with or without 1.5 mM STZ (24 h) ± 10 µM DXO (48 h) with 100 nM si-RNA. The nuclei were stained with Hoechst, living cells with calcein, and dead cells with ethidium homodimer-1 (EthD-1). **E**, Quantification of cell death (*n = 3* stainings from one islet isolation). Data are shown as individual values (mean ± SEM) with corresponding p-values and calculated as percentage of untreated Control islets. Statistical significance was determined by two-way ANOVA with Tukey's multiple comparisons (**E**). Scale bar: 50 µm in **A**, **D.**

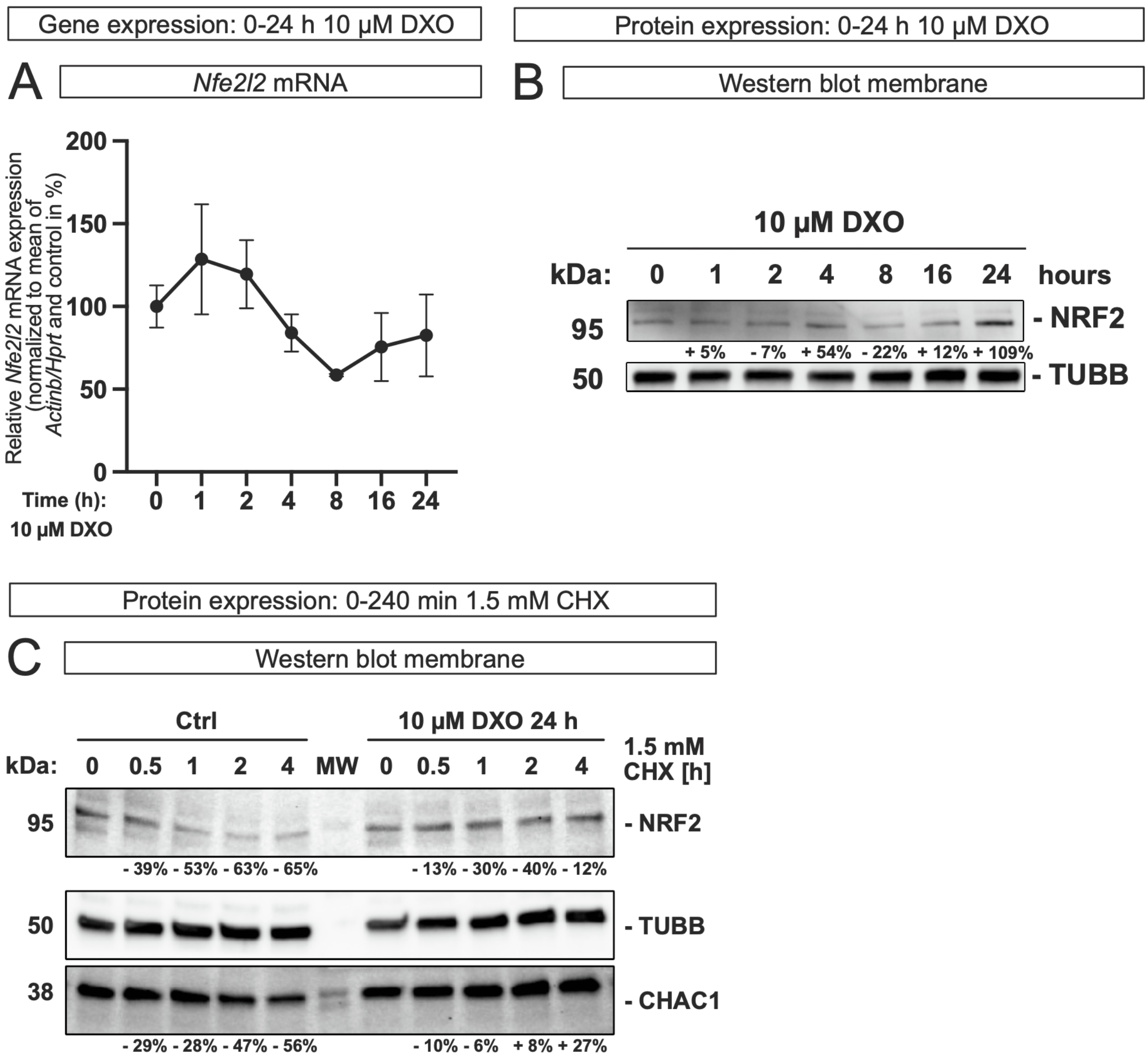

Figure S2. Time course of *Nfe2l2* and NRF2 expression after DXO treatment, and cycloheximide chase analysis of NRF2 and CHAC1 (related to Fig. 3) A, Relative *Nfe2l2* mRNA expression in mouse pancreatic islets following treatment with 10 µM DXO for 0, 1, 2, 4, 8, 16, or 24 h. Expression levels were normalized to the geometric mean of the housekeeping genes *Actb* and *Hprt* and are presented relative to the mean of untreated controls. *n* = 2 independent islet isolations. **B**, Immunoblot showing NRF2 and TUBB protein levels in mouse pancreatic islets treated with 10 µM DXO for 0, 1, 2, 4, 8, 16, or 24 h. The experiment was reproduced independently once with similar results. **C**, Immunoblot showing cycloheximide (CHX) chase of NRF2 and CHAC1 protein stability in mouse pancreatic islets pretreated with 10 µM DXO for 24 h, followed by treatment with 1.5 mM CHX for 0-4 h. For visualization purposes, brightness and contrast of the immunoblots in panels **B** and **C** were adjusted. *n = 1* islet isolation.

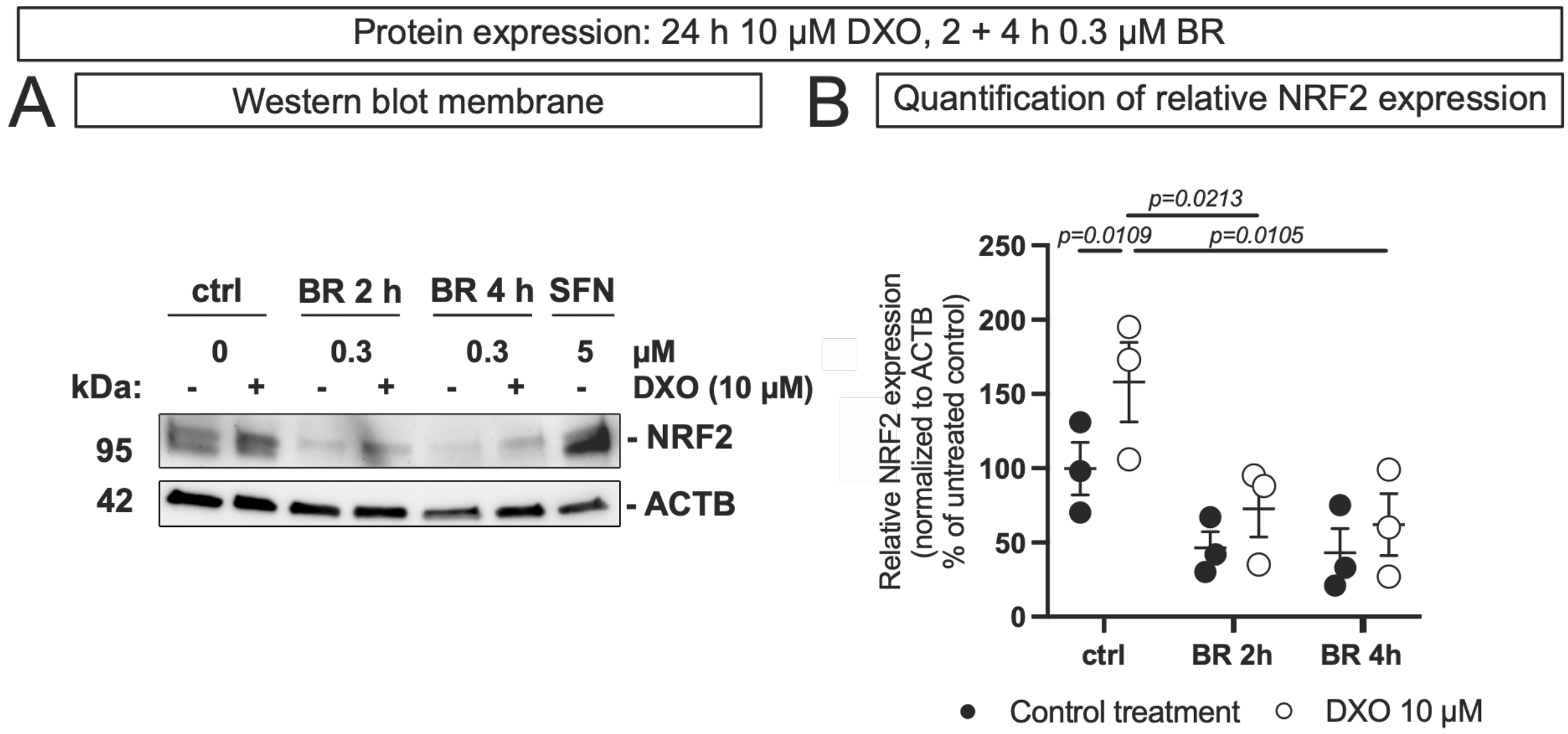

Figure S3. Establishment of brusatol (BR) as an NRF2 inhibitor in mouse pancreatic islets (related to Fig. 4). A, Immunoblot showing NRF2 and ACTB protein levels in mouse islets treated with 10 µM DXO for 24 h, with or without 0.3 µM brusatol (BR) for the final 2 or 4 h treatment. **B**, Quantification of NRF2 protein abundance normalized to ACTB relative to the mean of untreated controls. Data are presented as individual values with mean ± SEM; *n* = 3 independent islet isolations. For visualization purposes, brightness and contrast of the immunoblot in panel **A** were adjusted. Statistical significance in panel **B** was assessed by repeated-measures two-way ANOVA followed by Tukey's multiple-comparisons test.

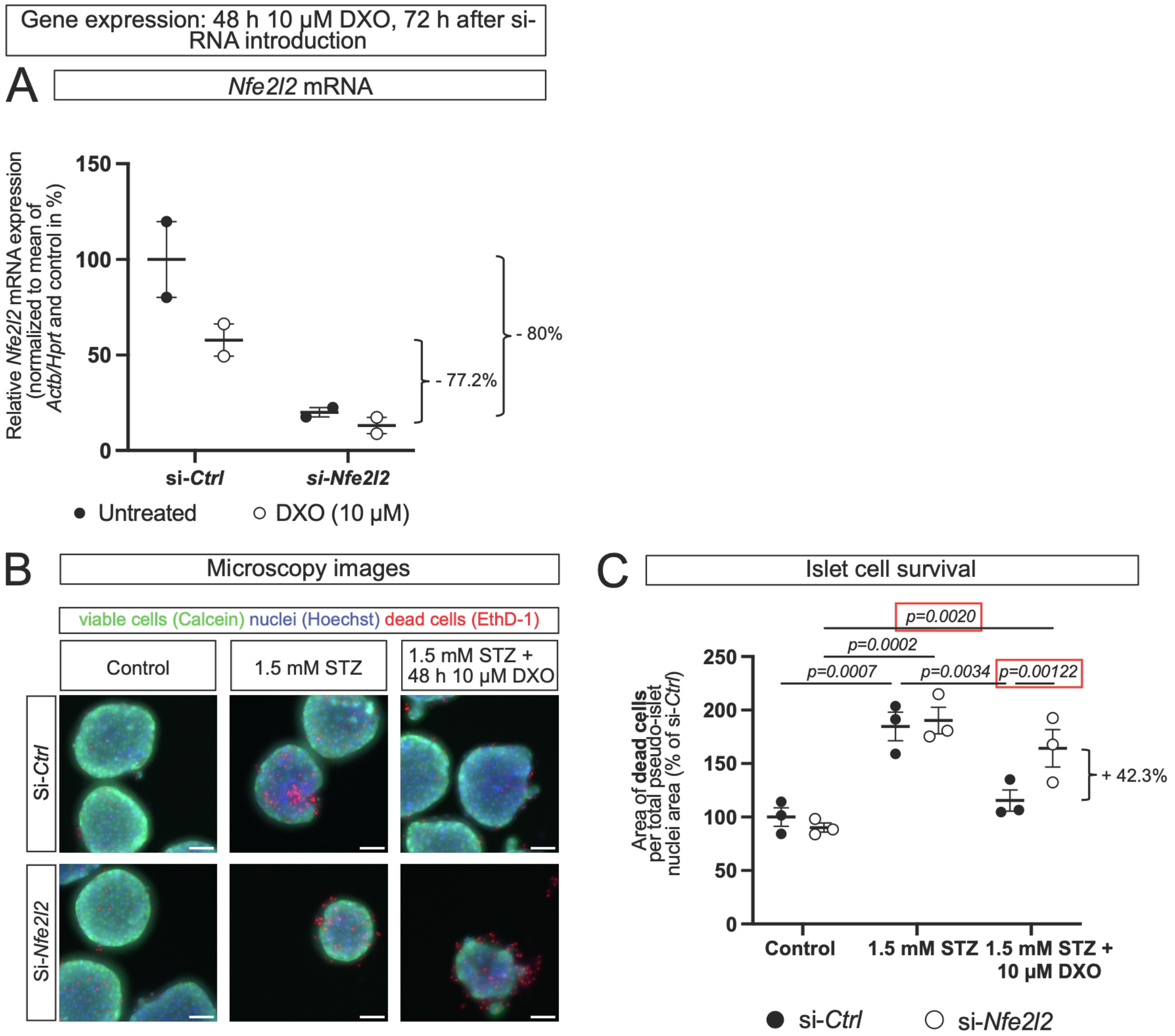

Figure S4. An efficient *Nfe2l2* knockdown reduces DXO-mediated islet protection against STZ toxicity (related to Fig. 5) A, Relative mRNA expression of *Nfe2l2* in mouse pancreatic pseudo-islets treated with 100 nM *Nfe2l2* siRNA after incubation with 10 µM DXO for 48 h. Values were normalized to the mean of housekeeping genes *Actb* and *Hprt* and the mean of the untreated control. n = 2. **B**, Images of live-dead imaging of si-*Ctrl* and si-*Nfe2l2* treated pseudo-islets with or without 1.5 mM STZ (24 h) ± 10 µM DXO (48 h) with 100 nM si-RNA. The nuclei were stained with Hoechst, living cells with calcein and dead cells with ethidium homodimer-1 (EthD-1). **C**, Quantification of cell death (n = 3 stainings from one islet isolation). Data are shown as individual values (mean ± SEM) with corresponding p-values and calculated as percentage of untreated control islets. Statistical significance was determined by two-way ANOVA with Tukey's multiple comparisons (**C**). Scale bar: 50 µm in **B**.

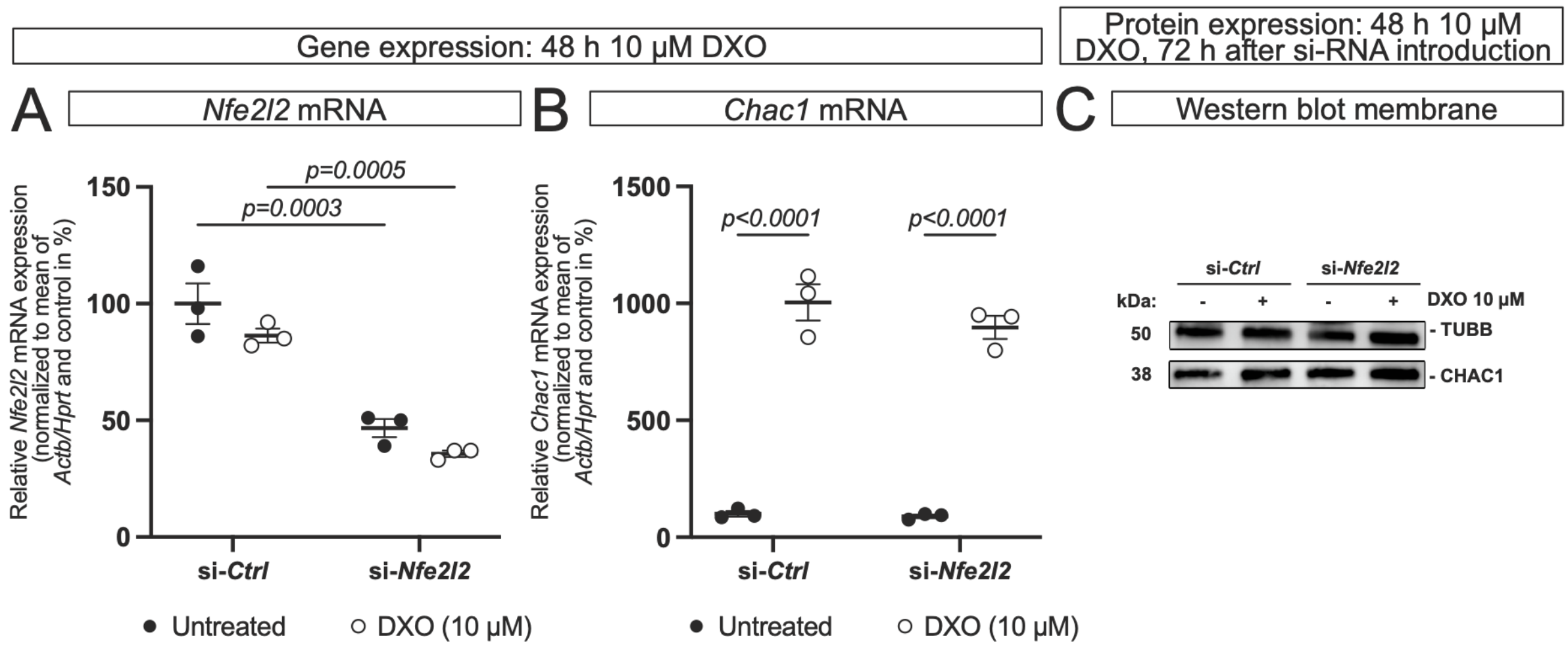

Figure S5. No detectable influence of NRF2 on CHAC1 expression in islets (related to Fig. 5). A, **B**, Relative mRNA expression of *Nfe2l2* and *Chac1* in mouse pancreatic pseudo-islets treated with 50 nM *Nfe2l2* siRNA after incubation with 10 µM DXO for 48 h. Values were normalized to the mean of housekeeping genes *Actb* and *Hprt* and the mean of the untreated control. *n = 3* independent islet isolations. **C**, TUBB/CHAC1 immunoblot of mouse pancreatic pseudo-islets treated with si-*Nfe2l2* KD and 10 µM DXO for 48 h. The contrast and brightness of **C** were adjusted for better visibility of the bands. *n = 1* islet isolation. Statistical significance was determined by two-way ANOVA with Tukey's multiple comparisons.

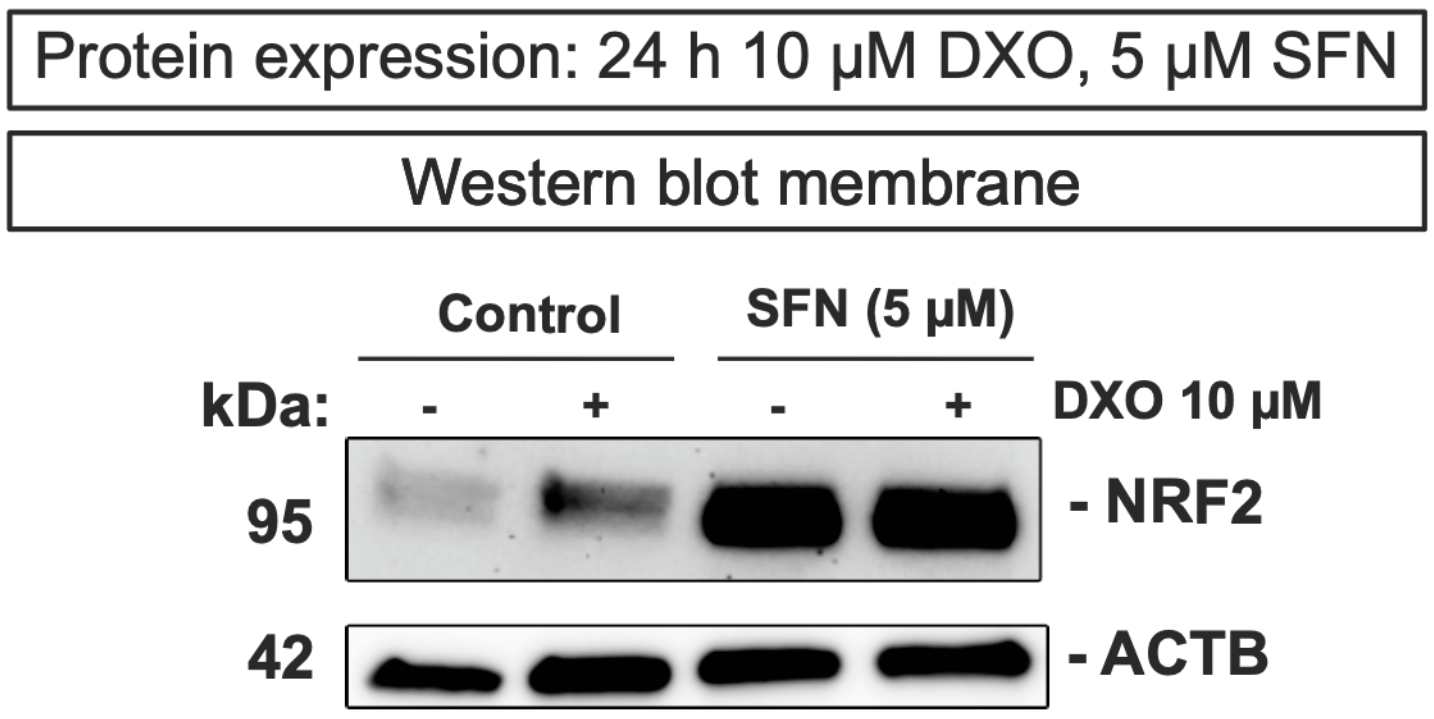

Figure S6. Establishment of Sulforaphane (SFN) as an NRF2 activator in mouse pancreatic islets (related to Fig. 6). NRF2/ACTB immunoblot of mouse pancreatic islets treated with 10 μM DXO for 24 h ± 5 μM SFN for 24 h. Reproduced in 2 independent experiments. The contrast and brightness were adjusted for better visibility of the bands.

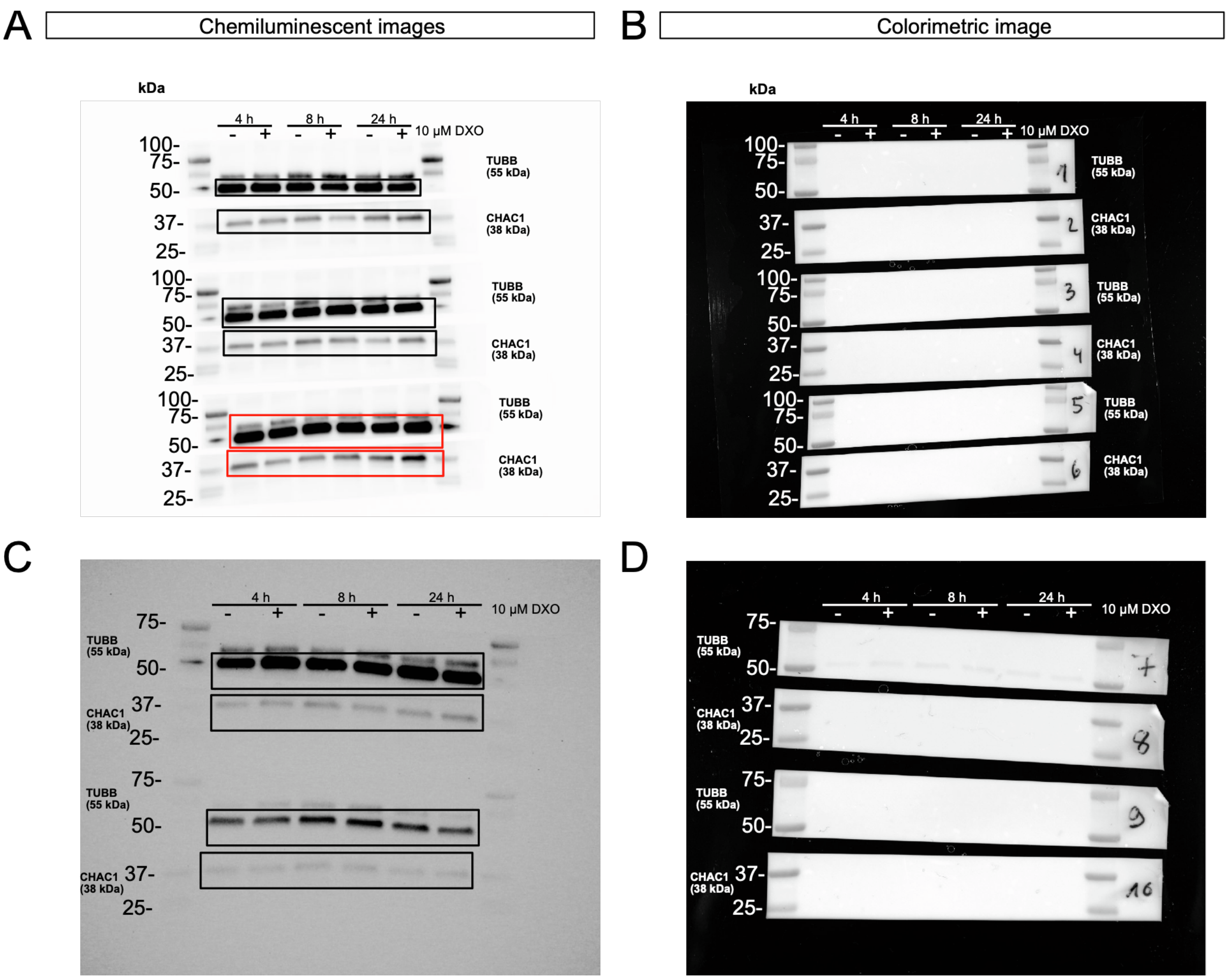

Data S1. Uncropped immunoblot images related to Fig. 1. Pancreatic islets were treated for 4, 8 or 24 h ±10 μM DXO. **A**, **C** show chemiluminescent images and **B**, **D** show the corresponding colorimetric images. Immunoblotting was performed using antibodies against CHAC1 and TUBB. Regions delineated by black boxes indicate the blots used for quantification; regions delineated by red boxes indicate those additionally shown as representative images in Fig. 1C. *n = 5* independent pancreatic islet isolations.

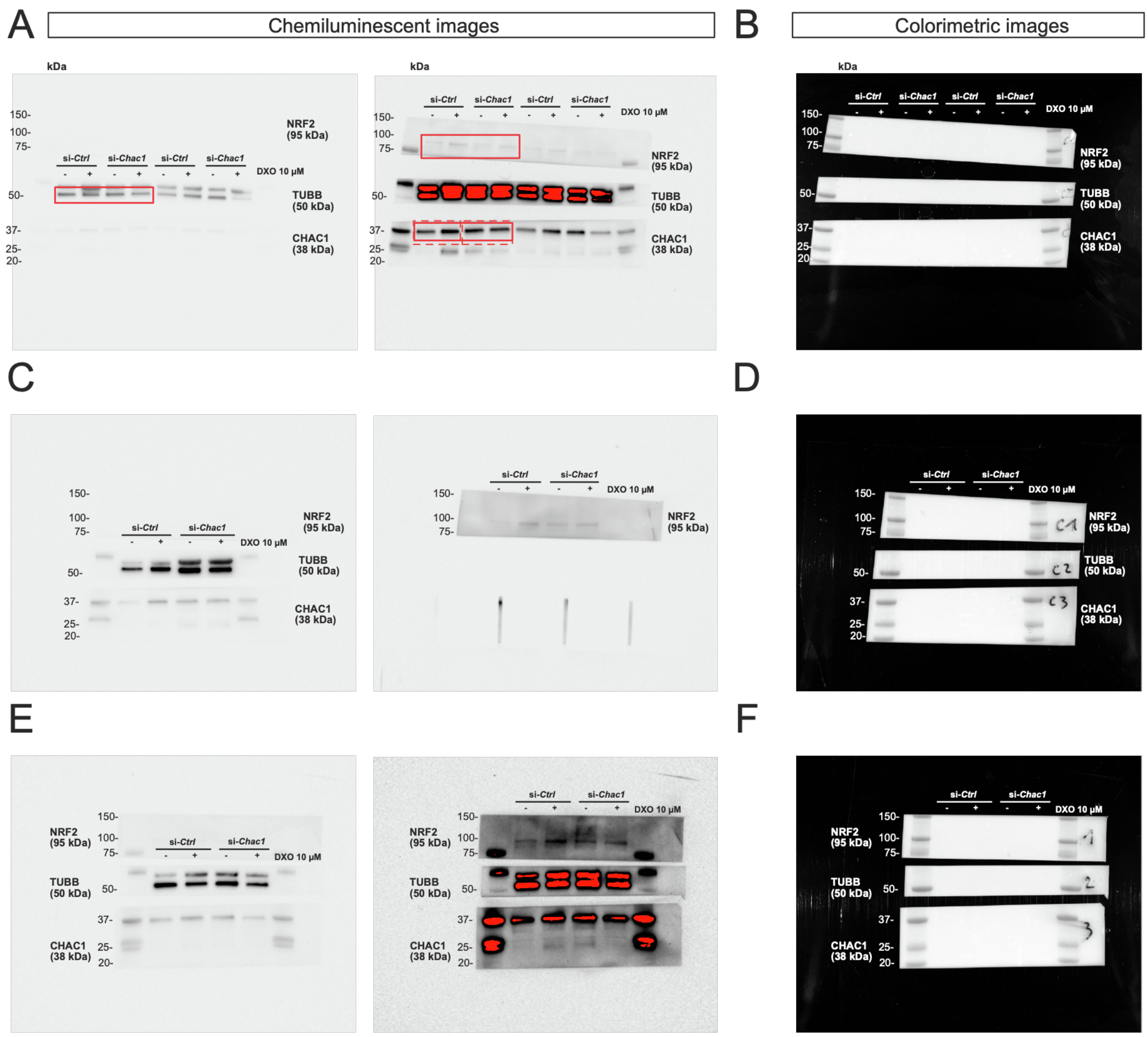

Data S2. Uncropped immunoblot images related to Fig. 2. Pancreatic pseudo-islets (si-*Ctrl* or si-*Chac1*) were treated for 48 h with 10 µM DXO. **A**, **C**, **E** show chemiluminescent images and **B**, **D**, **F** show the corresponding colorimetric images. Immunoblotting was performed using antibodies against NRF2, TUBB and CHAC1. Regions delineated by red boxes indicate bands shown as representative images in Fig. 2F. Dashed red boxes highlight the relative change in expression levels before and after DXO treatment in both conditions for CHAC1. *n = 4* independent pancreatic islet isolations.

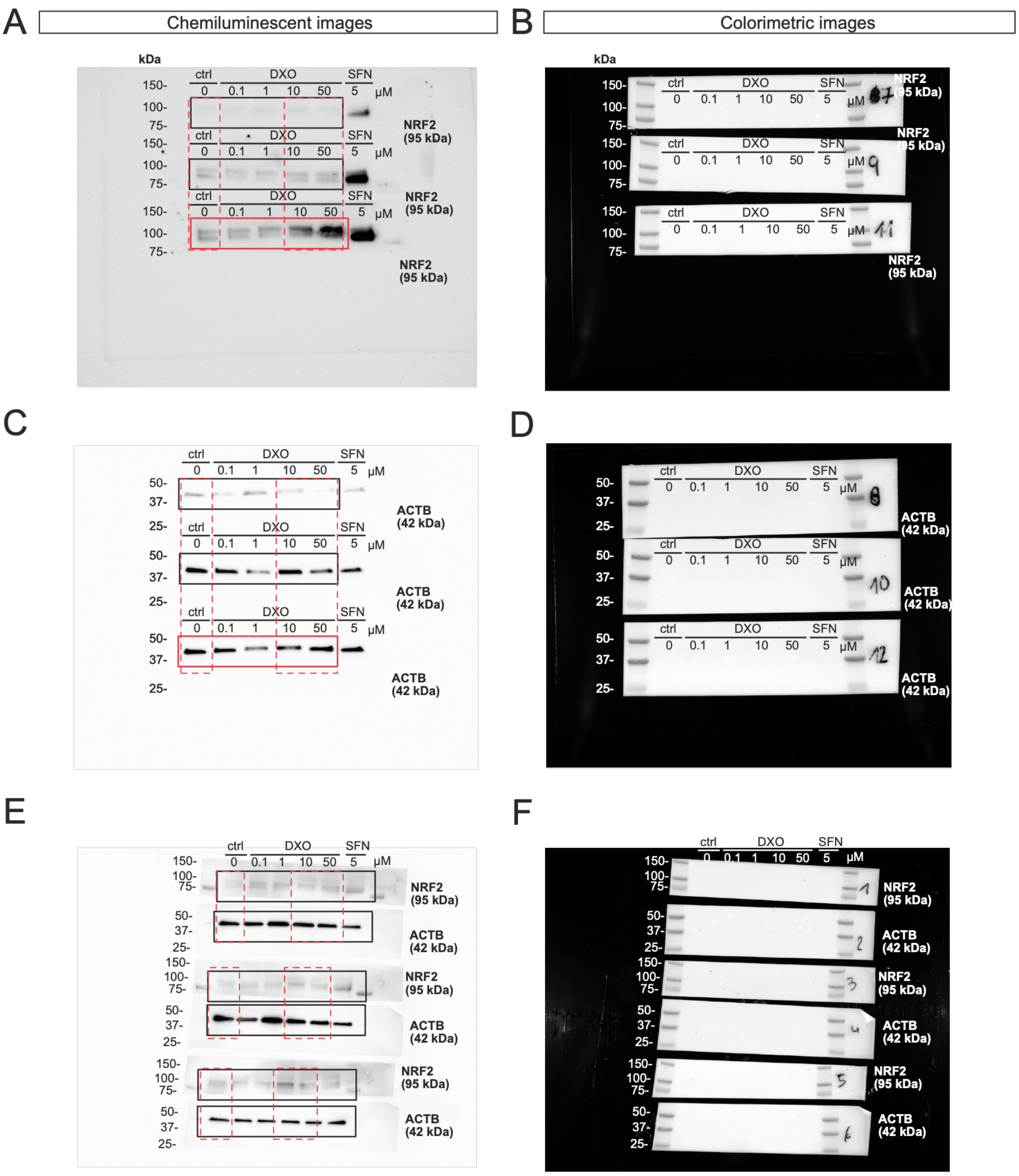

Data S3. Uncropped immunoblot images related to Fig. 3. Pancreatic islets were treated for 24 h with 0.1-50 µM DXO, or with 5 µM SFN for 24 h as a positive control for the antibody due to a generally low protein abundance of NRF2 in our samples. **A**, **C**, **E** show chemiluminescent images and **B**, **D**, **F** show the corresponding colorimetric images. Immunoblotting was performed using antibodies against NRF2 and ACTB. Regions delineated by black boxes indicate the blots used for quantification; regions delineated by red boxes indicate those additionally shown as representative images in Fig. 3B. *n = 6* independent pancreatic islet isolations.

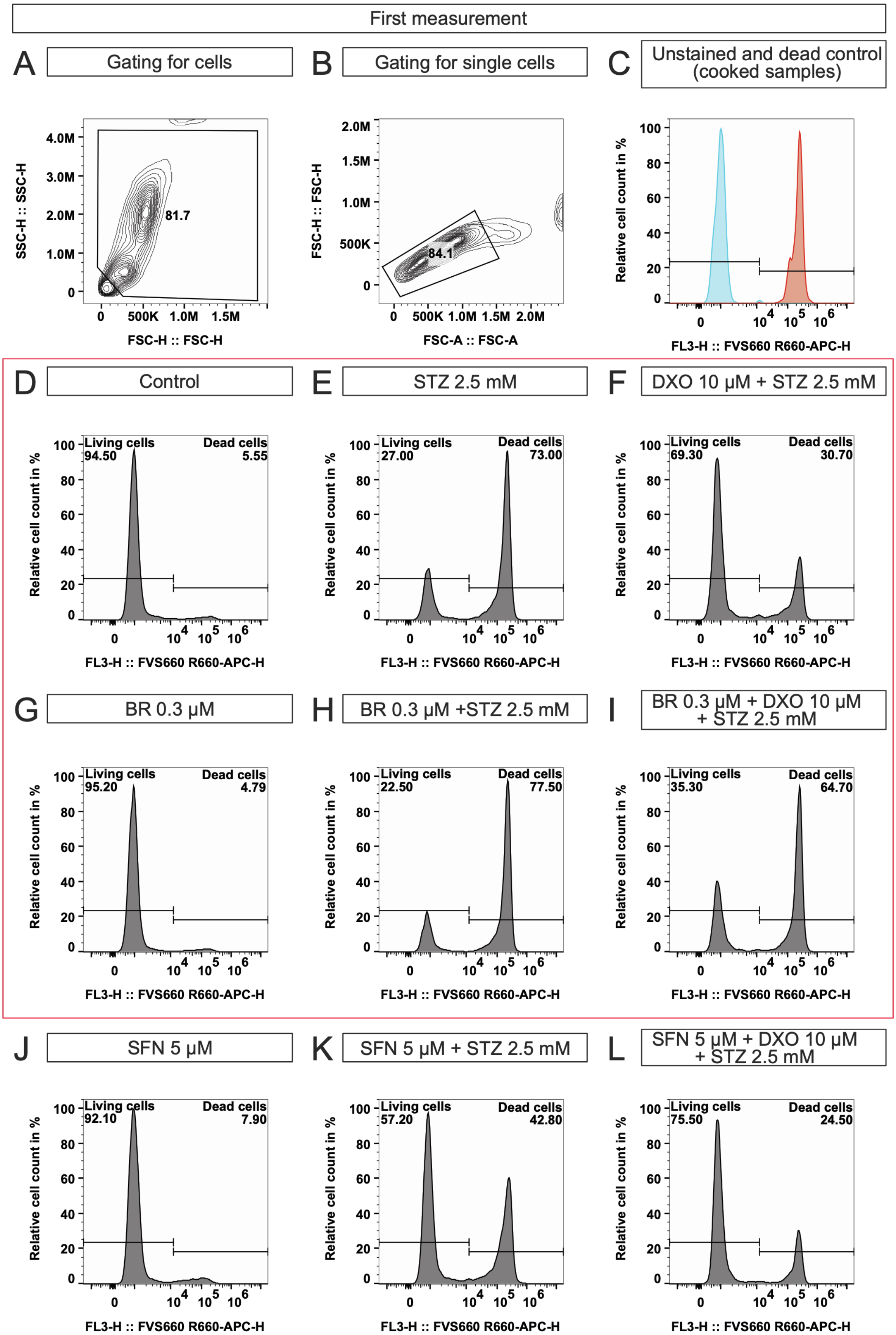
First measurement
A
Gating for cells
SSC-H :: SSC-H
FSC-H :: FSC-H
81.7
B
Gating for single cells
FSC-H :: FSC-H
FSC-A :: FSC-A
84.1
C
Unstained and dead control (cooked samples)
Relative cell count in %
FL3-H :: FVS660 R660-APC-H
D
Control
Living cells 94.50
Dead cells 5.55
E
STZ 2.5 mM
Living cells 27.00
Dead cells 73.00
F
DXO 10 μM + STZ 2.5 mM
Living cells 69.30
Dead cells 30.70
G
BR 0.3 μM
Living cells 95.20
Dead cells 4.79
H
BR 0.3 μM +STZ 2.5 mM
Living cells 22.50
Dead cells 77.50
I
BR 0.3 μM + DXO 10 μM + STZ 2.5 mM
Living cells 35.30
Dead cells 64.70
J
SFN 5 μM
Living cells 92.10
Dead cells 7.90
K
SFN 5 μM + STZ 2.5 mM
Living cells 57.20
Dead cells 42.80
L
SFN 5 μM + DXO 10 μM + STZ 2.5 mM
Living cells 75.50
Dead cells 24.50
Relative cell count in %
FL3-H :: FVS660 R660-APC-H

Data S4. Gating strategy and representative histograms of the first FACS measurement corresponding to Fig. 4 and 6. A, Initial gating of cells based on SSC-H *versus* FSC-H. **B**, Gating of single cells using FSC-A versus FSC-H. **C**, Relative cell counts (%) of unstained controls (blue) and dead-cell controls (red; heat-treated at 60 °C for 5 min). **D**–**L**, Representative histograms of untreated mouse pancreatic islets and islets exposed to 2.5 mM STZ for 24 h, either alone or with 0.3 μM BR (4 h pretreatment + 4 h cotreatment) or 5 μM SFN (48 h) ± 10 μM DXO (48 h). DMSO was included as a vehicle control. **D**, Control; **E**, STZ; **F**, STZ + DXO; **G**, BR; **H**, BR + STZ; **I**, BR + STZ + DXO; **J**, SFN; **K**, SFN + STZ; **L**, SFN + STZ + DXO. Red boxes indicate the histograms shown in the Fig. 4A-F.

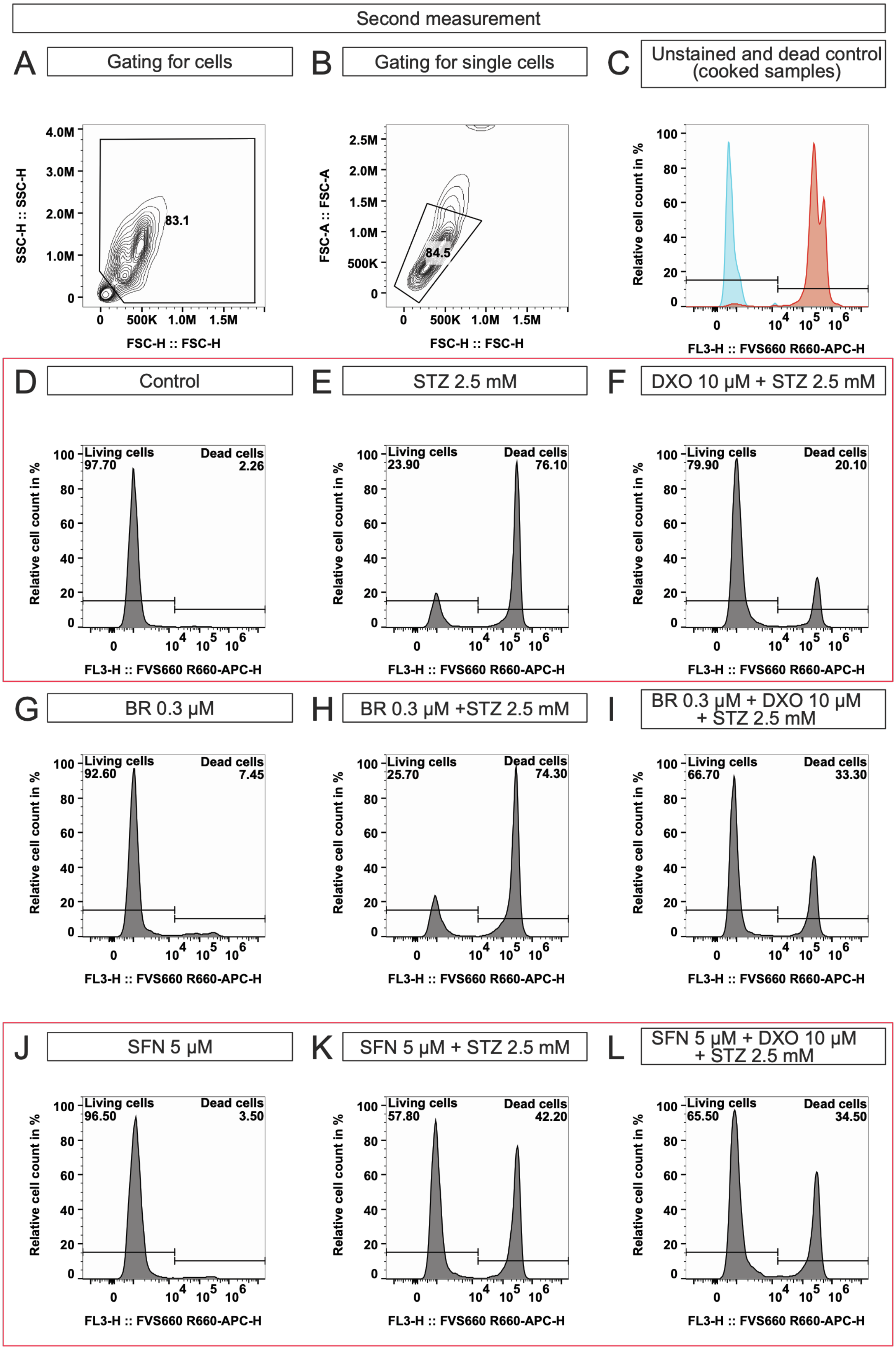

Second measurement
A
Gating for cells
B
Gating for single cells
C
Unstained and dead control (cooked samples)
SSC-H :: SSC-H
FSC-H :: FSC-H
83.1
FSC-A :: FSC-A
84.5
Relative cell count in %
FL3-H :: FVS660 R660-APC-H
D
Control
E
STZ 2.5 mM
F
DXO 10 μM + STZ 2.5 mM
Living cells 97.70
Dead cells 2.26
Living cells 23.90
Dead cells 76.10
Living cells 79.90
Dead cells 20.10
G
BR 0.3 μM
H
BR 0.3 μM +STZ 2.5 mM
I
BR 0.3 μM + DXO 10 μM + STZ 2.5 mM
Living cells 92.60
Dead cells 7.45
Living cells 25.70
Dead cells 74.30
Living cells 66.70
Dead cells 33.30
J
SFN 5 μM
K
SFN 5 μM + STZ 2.5 mM
L
SFN 5 μM + DXO 10 μM + STZ 2.5 mM
Living cells 96.50
Dead cells 3.50
Living cells 57.80
Dead cells 42.20
Living cells 65.50
Dead cells 34.50

Data S5. Gating strategy and representative histograms of the second FACS measurement corresponding to Fig. 4 and 6. A, Initial gating of cells based on side scatter height (SSC-H) *versus* forward scatter height (FSC-H). **B**, Gating of single cells using forward scatter area (FSC-A) versus FSC-H. **C**, Relative cell counts (%) of unstained controls (blue) and dead-cell controls (red; heat-treated at 60 °C for 5 min). **D**–**L**, Representative histograms of untreated mouse pancreatic islets and islets exposed to 2.5 mM STZ for 24 h, either alone or with 0.3 µM BR (4 h pretreatment + 4 h cotreatment) or 5 µM SFN (48 h) ± 10 µM DXO (48 h). DMSO was included as a vehicle control. **D**, Control; **E**, STZ; **F**, STZ + DXO; **G**, BR; **H**, BR + STZ; **I**, BR + STZ + DXO; **J**, SFN; **K**, SFN + STZ; **L**, SFN + STZ + DXO Red boxes indicate the histograms shown in Fig. 6A-F.

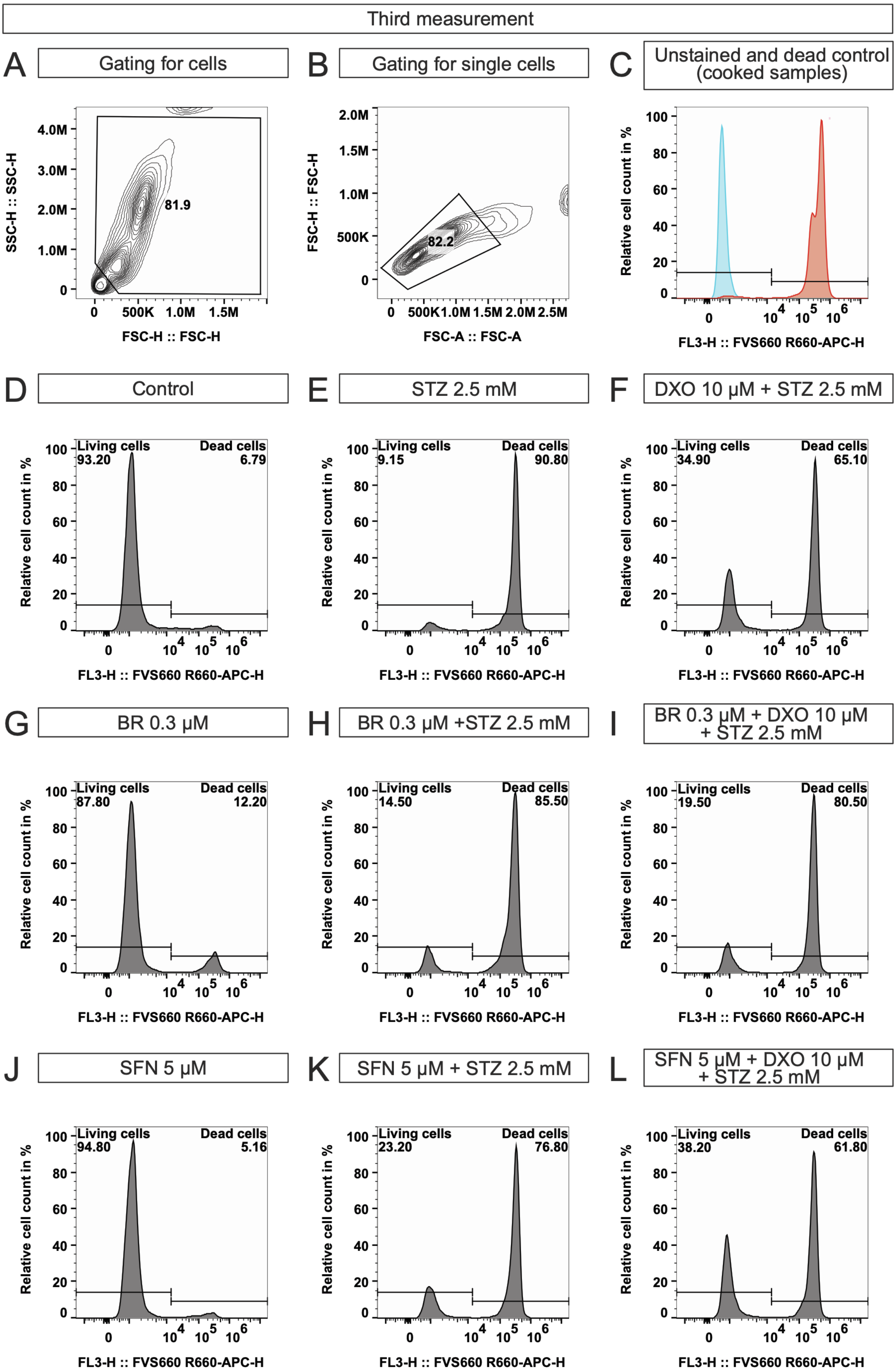

Third measurement
A
Gating for cells
81.9
SSC-H :: SSC-H
FSC-H :: FSC-H
B
Gating for single cells
82.2
FSC-H :: FSC-H
FSC-A :: FSC-A
C
Unstained and dead control (cooked samples)
Relative cell count in %
FL3-H :: FVS660 R660-APC-H
D
Control
Living cells 93.20
Dead cells 6.79
E
STZ 2.5 mM
Living cells 9.15
Dead cells 90.80
F
DXO 10 µM + STZ 2.5 mM
Living cells 34.90
Dead cells 65.10
G
BR 0.3 µM
Living cells 87.80
Dead cells 12.20
H
BR 0.3 µM +STZ 2.5 mM
Living cells 14.50
Dead cells 85.50
I
BR 0.3 µM + DXO 10 µM + STZ 2.5 mM
Living cells 19.50
Dead cells 80.50
J
SFN 5 µM
Living cells 94.80
Dead cells 5.16
K
SFN 5 µM + STZ 2.5 mM
Living cells 23.20
Dead cells 76.80
L
SFN 5 µM + DXO 10 µM + STZ 2.5 mM
Living cells 38.20
Dead cells 61.80

Data S6. Gating strategy and representative histograms of the third FACS measurement corresponding to Fig. 4 and 6. A, Initial gating of cells based on SSC-H versus FSC-H. **B**, Gating of single cells using FSC-A versus FSC-H. **C**, Relative cell counts (%) of unstained controls (blue) and dead-cell controls (red; heat-treated at 60 °C for 5 min). **D**–**L**, Representative histograms of untreated mouse pancreatic islets and islets exposed to 2.5 mM STZ for 24 h, either alone or with 0.3 µM BR (4 h pretreatment + 4 h cotreatment) or 5 µM SFN (48 h) ± 10 µM DXO (48 h). DMSO was included as a vehicle control. **D**, Control; **E**, STZ; **F**, STZ + DXO; **G**, BR; **H**, BR + STZ; **I**, BR + STZ + DXO; **J**, SFN; **K**, SFN + STZ; **L**, SFN + STZ + DXO

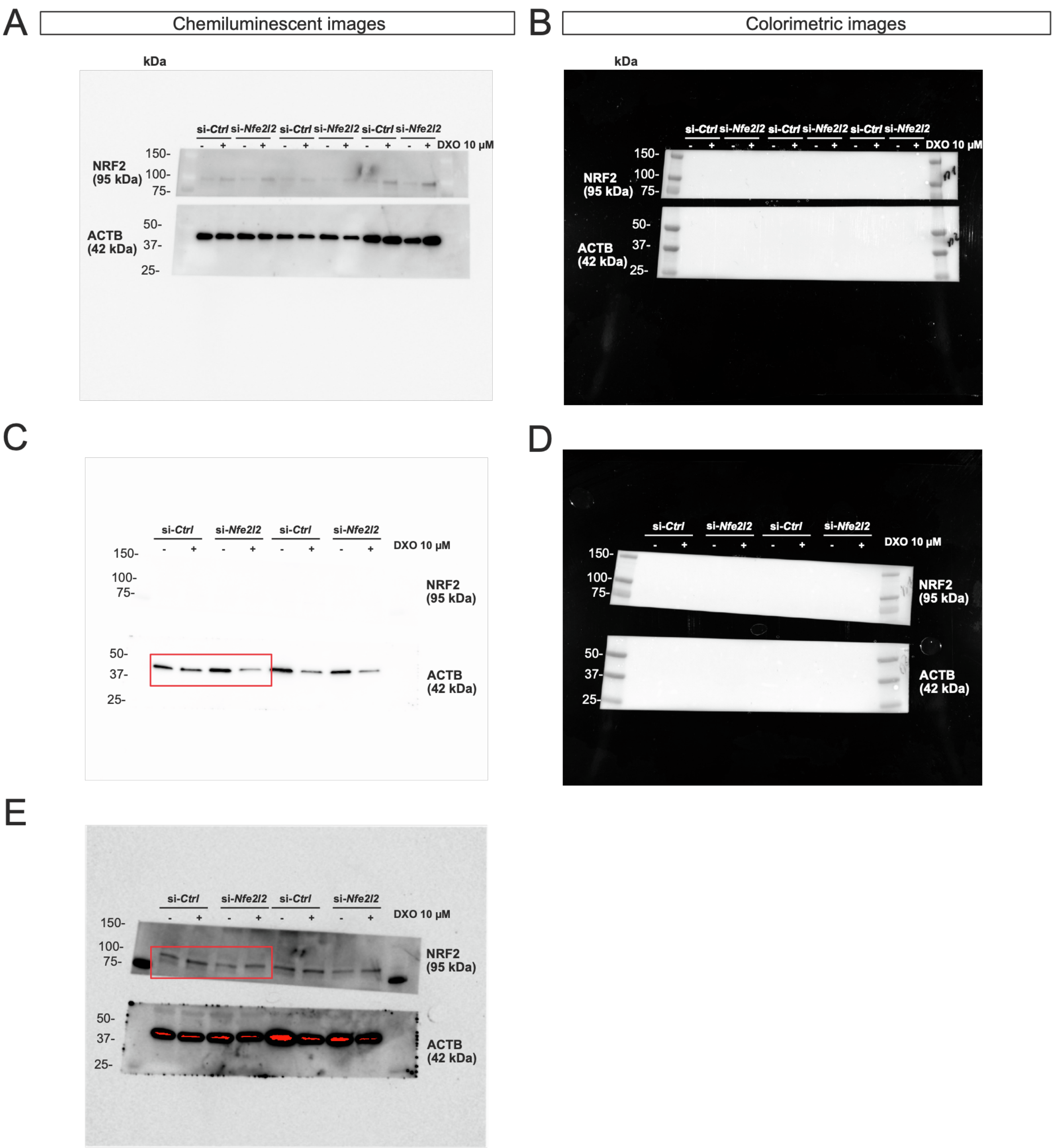

Data S7. Uncropped immunoblot images related to Fig. 5. Pancreatic pseudo-islets (*Ctrl* or *Nfe2l2* siRNA-treated) were incubated for 48 h with 10 μM DXO. **A**, **C**, **E** show chemiluminescent images and **B**, **D** show the corresponding colorimetric images. Immunoblotting was performed using antibodies against NRF2 and ACTB. Regions delineated by red boxes indicate bands shown as representative images in Fig. 5D. *n* = *5* independent pancreatic islet isolations.

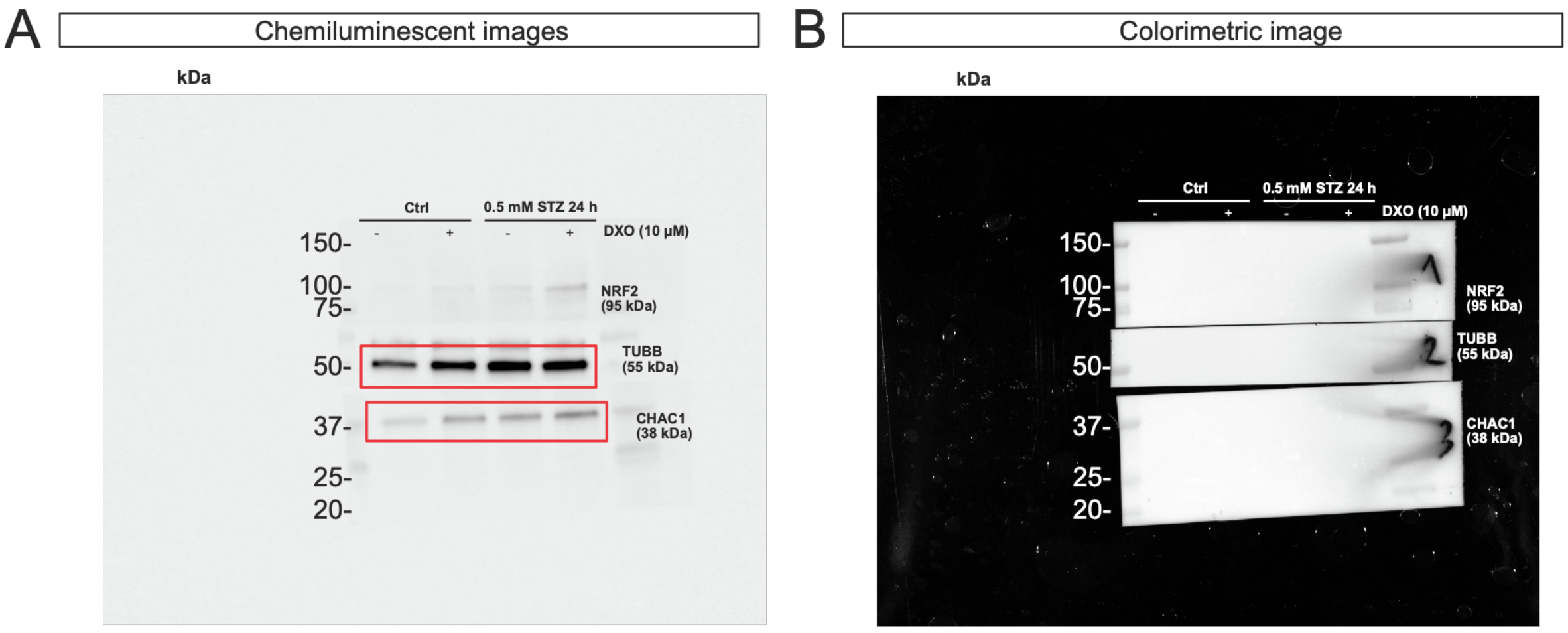

Data S8. Uncropped immunoblot images related to Fig. S1. Pancreatic islets were incubated for 24 h ± 10 µM DXO ± 0.5 mM STZ. **A**, shows the corresponding chemiluminescent image and **B**, the colorimetric image. Immunoblotting was performed using antibodies against NRF2, CHAC1 and TUBB. Regions delineated by red boxes indicate bands shown as representative images in Fig. S1B. *n = 1* islet isolation.

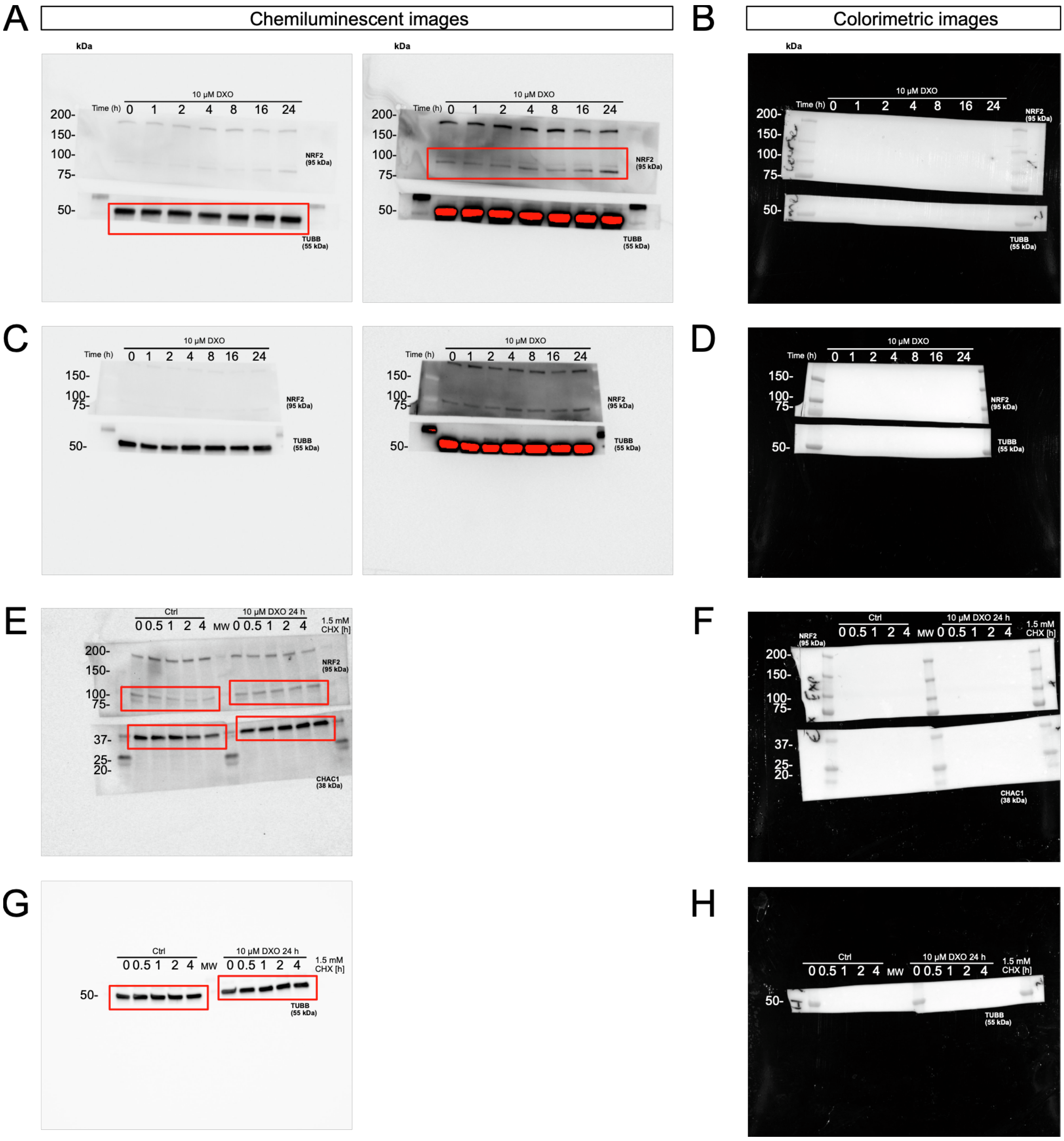

Data S9. Uncropped immunoblot images related to Fig. S2. Pancreatic islets were incubated for 0, 1, 2, 4, 8, 16, 24 h with 10 μM DXO in **A**-**D**, or for 24 h with 10 μM DXO followed by 1.5 mM CHX for 0, 0.5, 1, 2, 4 h (**E**-**H**). **A**, **C**, **E**, **G**, show chemiluminescent images and **B**, **D**, **F**, **H** show the corresponding colorimetric images. Immunoblotting was performed using antibodies against CHAC1, NRF2 and TUBB. Regions delineated by red boxes indicate bands shown as representative images in Fig. S2B,C. *n* = 2 independent pancreatic islet isolations for **A**-**D**. *n* = *1* islet isolation for **E**-**H**.

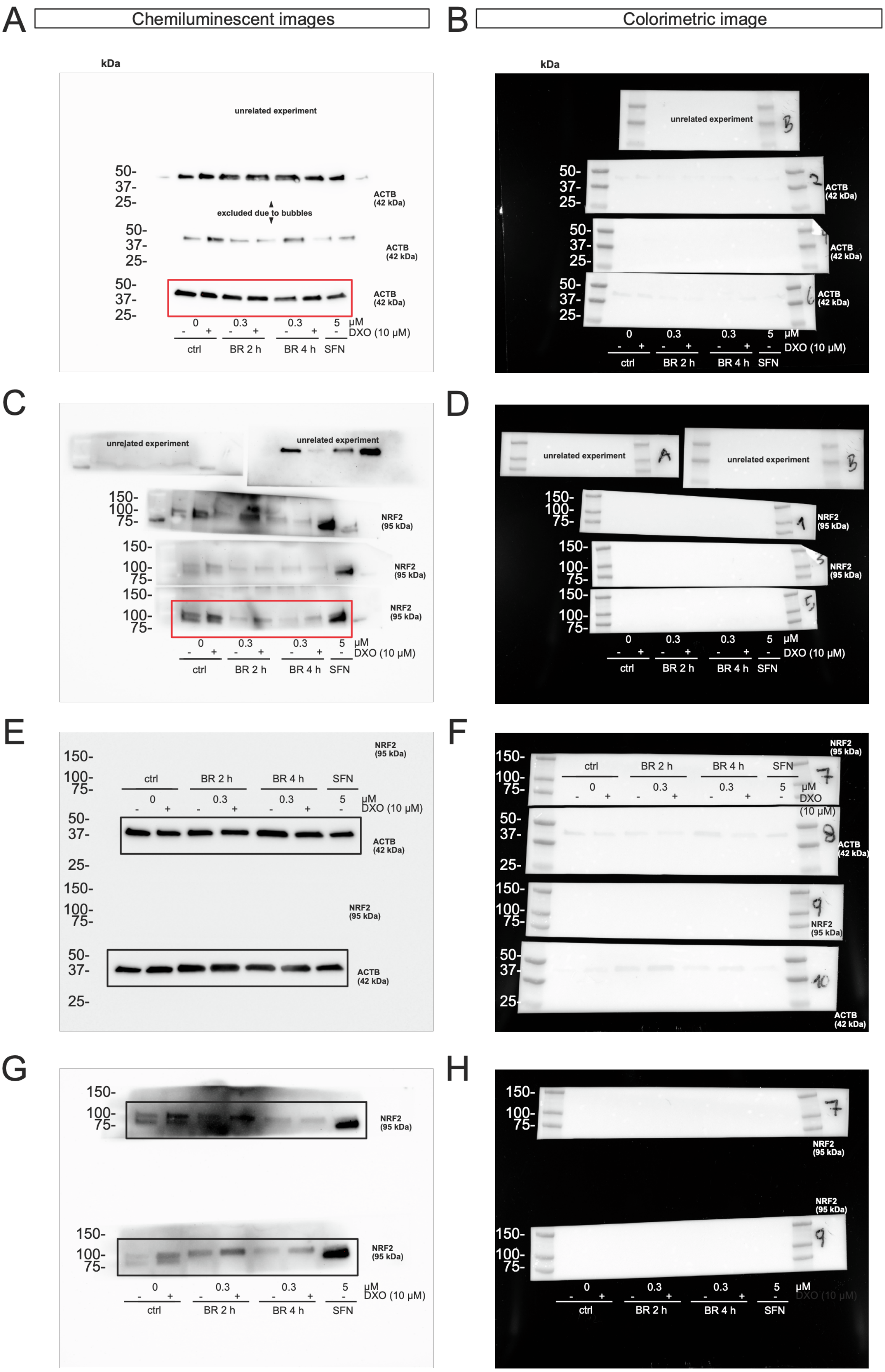
A
Chemiluminescent images
B
Colorimetric image
kDa
unrelated experiment
excluded due to bubbles
50-
37-
25-
ACTB (42 kDa)
0
0.3
0.3
5
µM
DXO (10 µM)
ctrl
BR 2 h
BR 4 h
SFN
C
D
150-
100-
75-
NRF2 (95 kDa)
E
F
G
H

Data S10. Uncropped immunoblot images related to Fig. S3. Pancreatic islets were treated with 10 µM DXO for 24 h ± 0.3 µM BR for 2 or 4 h and 5 µM SFN for 24 h as a positive control. **A**, **C**, **E**, **G** show chemiluminescent images and **B**, **D**, **F**, **H** show the corresponding colorimetric images. Immunoblotting was performed using antibodies against NRF2 and ACTB. Regions delineated by red boxes indicate bands shown as representative images in Fig. S3A, black boxes indicate blots used for analysis. *n = 3* independent pancreatic islet isolations.

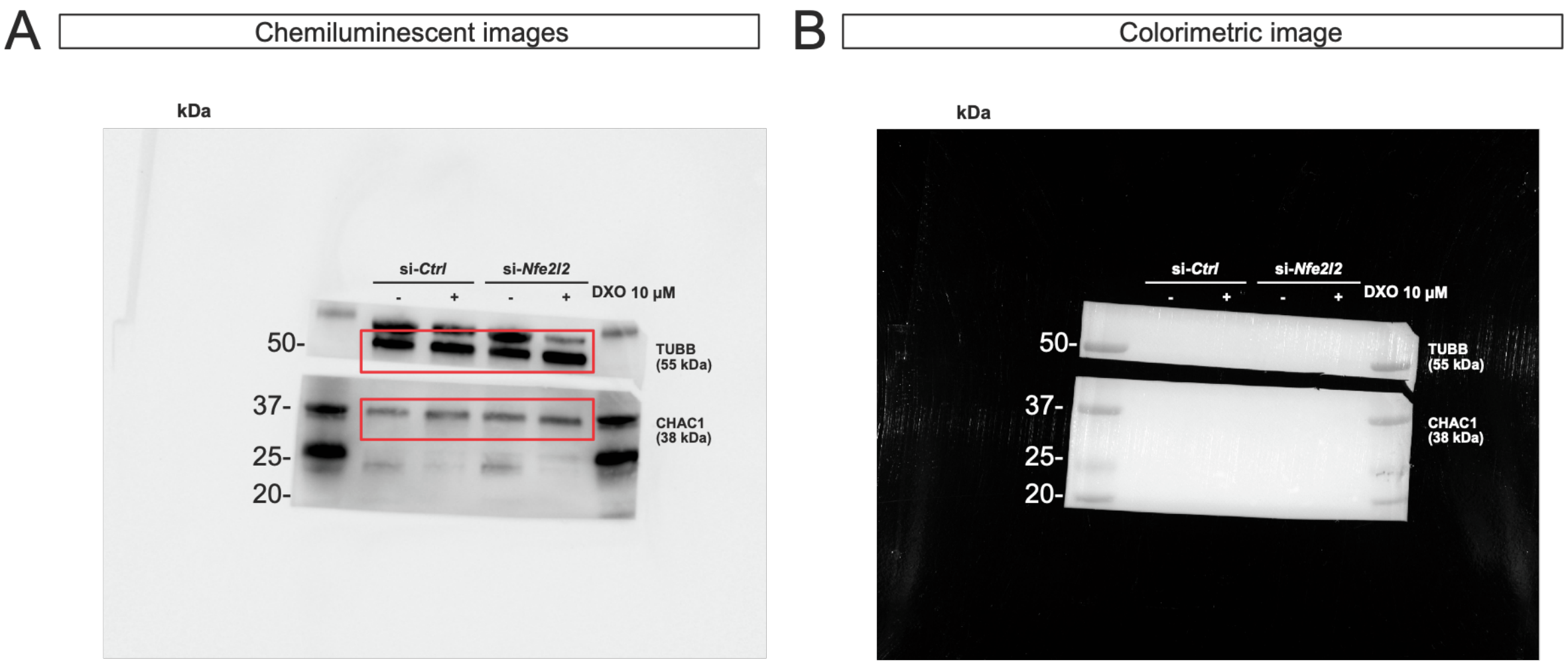

Data S11. Uncropped immunoblot images related to Fig. S5. Pancreatic pseudo-islets (si-*Ctrl* or si-*Nfe2l2* siRNA treated) were incubated for 48 h with 10 µM DXO. **A**, shows the corresponding chemiluminescent image and **B** shows the corresponding colorimetric image. Immunoblotting was performed using antibodies against TUBB and CHAC1. Regions delineated by red boxes indicate bands shown as representative images in Fig. S5C. *n = 1* islet isolation.

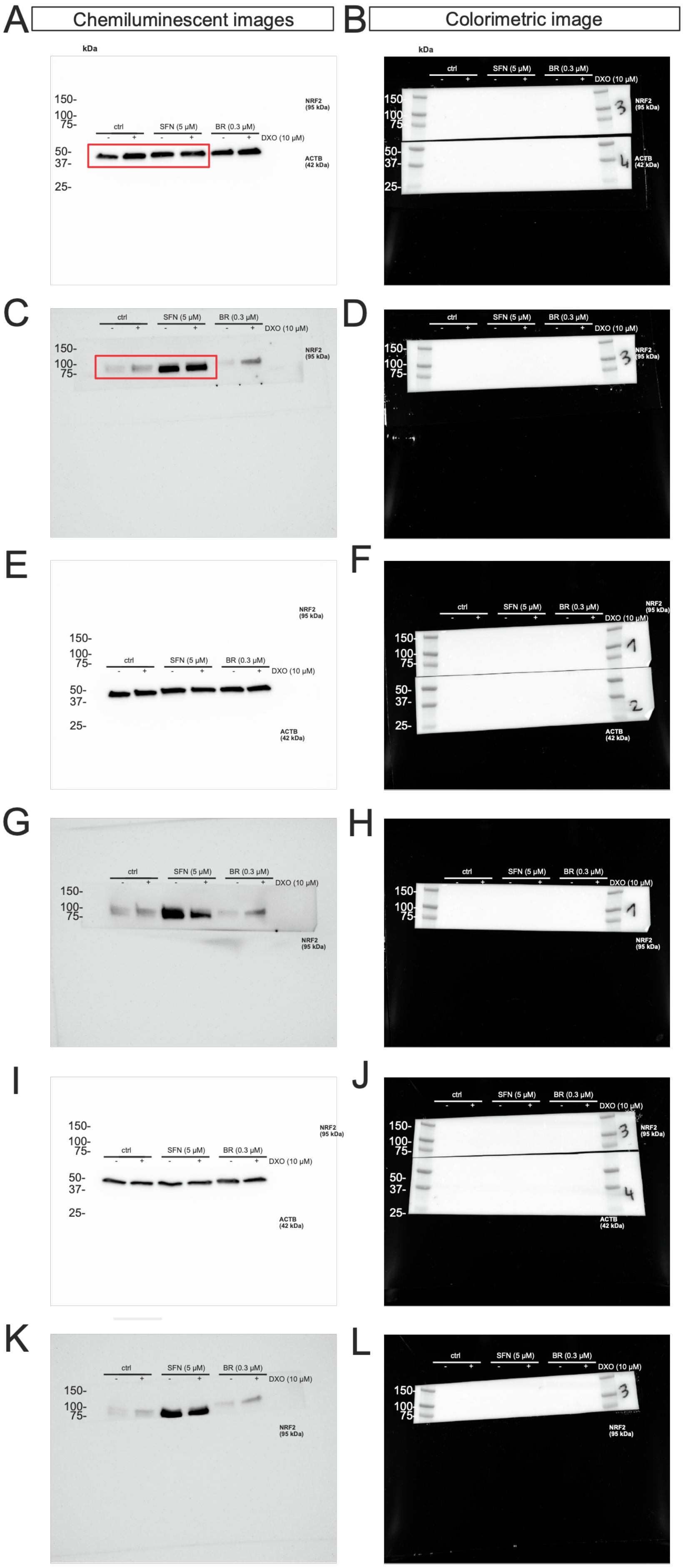
A
Chemiluminescent images
B
Colorimetric image
kDa
150-
100-
75-
50-
37-
25-
ctrl
SFN (5 µM)
BR (0.3 µM)
DXO (10 µM)
NRF2 (95 kDa)
ACTB (42 kDa)
C
D
E
F
G
H
I
J
K
L

Data S12. Uncropped immunoblot images related to Fig. S6. Pancreatic islets were treated with 10 μM DXO for 24 h ± 0.3 μM BR for 24 h and ± 5 μM SFN for 24 h. **A**, **C**, **E**, **G**, **I**, **K** show chemiluminescent images and **B**, **D**, **F**, **H**, **J**, **L** show the corresponding colorimetric images. Immunoblotting was performed using antibodies against NRF2 and ACTB. Regions delineated by red boxes indicate bands shown as representative images in Fig. S6. $n = 3$ independent pancreatic islet isolations.

Table S1: List of selected genes

GENE SYMBOL	FULL GENE NAME
Abcc1	*ATP Binding Cassette Subfamily C Member 1*
Abcc2	*ATP Binding Cassette Subfamily C Member 2*
Akr1b3	*Aldo-Keto Reductase Family 1 Member B3*
Akr1c14	*Aldo-Keto Reductase Family 1 Member C14*
Akr7a5	*Aldo-Keto Reductase Family 7 Member A5*
Cbr1	*Carbonyl Reductase 1*
Chac1	*ChaC Glutathione Specific Gamma-Glutamylcyclotransferase 1*
Ephx1	*Epoxide Hydrolase 1*
Ets1	*ETS Proto-Oncogene 1, Transcription Factor*
Fth1	*Ferritin Heavy Chain 1*
Ftl1	*Ferritin Light Chain 1*
Gclc	*Glutamate–Cysteine Ligase Catalytic Subunit*
Gclm	*Glutamate–Cysteine Ligase Modifier Subunit*
Gnai2	*G Protein Subunit Alpha i2*
Gpx2	*Glutathione Peroxidase 2*
Gsr	*Glutathione-Disulfide Reductase*
Gsta1	*Glutathione S-Transferase Alpha 1*
Gsta3	*Glutathione S-Transferase Alpha 3*
Gstm4	*Glutathione S-Transferase Mu 4*
Gstm5	*Glutathione S-Transferase Mu 5*
Gstp2	*Glutathione S-Transferase Pi 2*
Hmox1	*Heme Oxygenase 1*
Mafg	*MAF BZIP Transcription Factor G*
Mapk14	*Mitogen-Activated Protein Kinase 14*
Mgst1	*Microsomal Glutathione S-Transferase 1*
Nfe2l2	*Nuclear Factor, Erythroid 2 Like 2*
Nqo1	*NAD(P)H Quinone Dehydrogenase 1*
Nqo2	*NAD(P)H Quinone Dehydrogenase 2*
Prdx1	*Peroxiredoxin 1*
Slc7a1	*Solute Carrier Family 7 Member 1*
Slc7a11	*Solute Carrier Family 7 Member 11*
Slc7a2	*Solute Carrier Family 7 Member 2*
Slc7a3	*Solute Carrier Family 7 Member 3*
Slc7a4	*Solute Carrier Family 7 Member 4*
Sod1	*Superoxide Dismutase 1*
Sqstm1	*Sequestosome 1*

Txn1	*Thioredoxin 1*
Txnrd1	*Thioredoxin Reductase 1*
Usp14	*Ubiquitin Specific Peptidase 14*

Table S2: List of Antibodies

REAGENT or RESOURCE	SOURCE	IDENTIFIER
Antibodies		
Mouse anti-ACTB, monoclonal	Sigma Aldrich	Cat#A5441; RRID:AB_476744
Rabbit anti-NRF2, polyclonal	ProteinTech	Cat#80593-1-RR; RRID:AB_2918904
Rabbit anti-CHAC1, polyclonal	ProteinTech	Cat#15207-1-AP; RRID:AB_2878118
Rabbit anti-TUBB, polyclonal	Abcam	Cat#ab6046; RRID:AB_2210370
Hourse anti-Mouse IgG HRP, polyclonal	Cell signaling	Cat#7076; RRID:AB_330924
Goat anti-Rabbit IgG HRP, polyclonal	Cell signaling	Cat#7074; RRID:AB_2099233

Table S3: List of Oligonucleotides

REAGENT or RESOURCE	SOURCE	IDENTIFIER
Oligonucleotides		
ON-TARGETplus Mouse *Nfe2l2* siRNA – SMARTpool ACUCAAAUCCCACCUUAAA, UGGAGUAAGUCGAGAAGUG, CAUGUUACGUGAUGAGGAU, GGACAGCAAUUACCAUUUU<br><br>Mouse *Nfe2l2* siRNA GCAUGUUACGUGAUGAGGAtt	Horizon Discovery<br><br>ThermoFischer	Cat#L-040766-00-0006;<br><br>Cat#4390771 – s70521
ON-TARGETplus Mouse *Chac1* siRNA – SMARTpool CGACAAGAUGCCUGGCCGA, CAGAGAAGGAGGCGAAGUA, UUGAGUACUUAUUGCGUUU, GUACAGGGCUUGGGUAAGU<br><br>Mouse *Chac1* siRNA CUUGAGUACUUAUUGCGUUtt	Horizon Discovery<br><br>Thermo Fischer	Cat#L-063606-01-0005;<br><br>Cat#4390771 – s87391
ON-TARGETplus Non-targeting Pool UGGUUUACAUGUCGACUAA, UGGUUUACAUGUUUUCUGA, UGGUUUACAUGUUUUCCUA, UGGUUUACAUGUUGUGUGA	Horizon Discovery	Cat#D-001810-10-50
Mouse *Beta-actin*: CTAAGGCCAACCGTGAAAAG; ACCAGAGGCATACAGGGACA	Eurogentec	N/A
Mouse *Hprt*: GCTGGTGAAAAGGACCTCT; CACAGGACTAGAACACCTGC	Eurogentec	N/A
Mouse *Nfe2l2*: TAGATGACCATGAGTCGCTTGC; GCCAAACTTGCTCCATGTCC	Eurogentec	N/A
Mouse *Chac1*: CTGTGGATTTTCGGGTACGG; CCCCTATGGAAGGTGTCTCC	Eurogentec	N/A
